# Structural basis for sensitivity and acquired resistance of fungal cap guanine-N7 methyltransferases to the antifungal antibiotic sinefungin

**DOI:** 10.1093/nar/gkaf538

**Published:** 2025-07-19

**Authors:** Daniel J Nilson, Beate Schwer, Steven C Almo, Stewart Shuman, Agnidipta Ghosh

**Affiliations:** Department of Biochemistry, Albert Einstein College of Medicine, 1300 Morris Park Avenue, Bronx, New York, NY 10461, United States; Department of Microbiology and Immunology, Weill Cornell Medical College, New York, NY 10021, United States; Department of Biochemistry, Albert Einstein College of Medicine, 1300 Morris Park Avenue, Bronx, New York, NY 10461, United States; Molecular Biology Program, Memorial Sloan Kettering Cancer Center, New York, NY 10021, United States; Department of Biochemistry, Albert Einstein College of Medicine, 1300 Morris Park Avenue, Bronx, New York, NY 10461, United States

## Abstract

The essential enzyme messenger RNA (mRNA) (guanine-N7) methyltransferase catalyzes *S*-adenosylmethionine (SAM)-dependent conversion of GpppRNA ends to the m^7^GpppRNA cap structure characteristic of eukaryal mRNAs. The antibiotic sinefungin (SFG) is a SAM analog in which the S-CH_3_ sulfonium moiety of SAM is replaced by a C-NH_2_ amine. Available evidence indicates that the antifungal activity of SFG is exerted via inhibition of fungal cap methyltransferase Abd1. Here we report that recombinant *Kluyveromyces lactis* and *Saccharomyces cerevisiae* Abd1 are 240-fold and 485-fold more sensitive to inhibition by SFG than by the reaction product S-adenosylhomocysteine (SAH). Crystal structures of *K. lactis* and *S. cerevisiae* Abd1 as binary complexes with SAH or SFG and ternary complexes with GTP•SFG highlight how SFG makes two hydrogen bonds from its C-NH_2_ amine to the guanine-O6 and -N7 atoms of GTP that account for its higher affinity vis-à-vis SAH and SAM. Through a genetic screen to isolate SFG-resistant *S. cerevisiae* strains, a conserved tyrosine (Tyr416) that interacts with the cap guanine in Abd1 was identified as a key determinant of SFG potency. Tyr416 Abd1 variants confer SFG resistance *in vitro* by weakening cap-assisted SFG interactions with Abd1. Our study illuminates the basis for the exquisite SFG sensitivity of fungal cap methyltransferases.

## Graphical Abstract

**Graphical Abstract:**
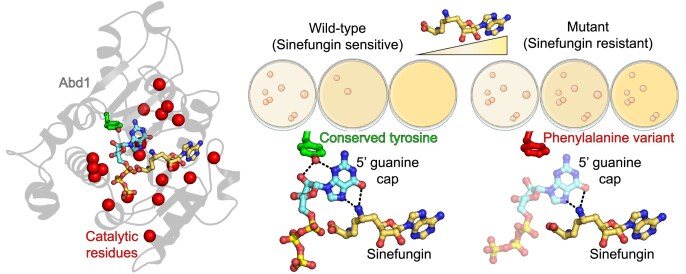

## Introduction

Nuclear pre-messenger RNAs (pre-mRNAs) and many viral mRNAs acquire an m^7^GpppN cap structure via a three-step pathway whereby (i) RNA triphosphatase hydrolyzes the 5′-triphosphate end of the nascent pre-mRNA to a diphosphate; (ii) RNA guanylyltransferase transfers GMP from GTP to the RNA 5′-diphosphate end to yield a GpppRNA cap; and (iii) RNA (guanine-N7) methyltransferase transfers a methyl group from *S*-adenosylmethionine (SAM) to GpppRNA to form an m^7^GpppRNA cap and *S*-adenosylhomocysteine (SAH) [1].

The capping pathway is an attractive target for antiviral, antifungal, and antiprotozoal drug discovery [2]. Inhibition of pathogen-encoded RNA (guanine-N7) methyltransferases has been suggested as an anti-infective strategy based on the findings that (i) inhibitors of cellular SAH hydrolase that elevate the level of SAH interdict replication of many viruses that encode their own capping enzymes [3]; (ii) the natural product antibiotic sinefungin (SFG), in which the S-CH_3_ sulfonium moiety of SAM is replaced by a C-NH_2_ amine, inhibits the growth and replication of a wide range of viruses, fungi, and protozoan parasites [2]; and (iii) SFG displays *in vivo* selectivity for inhibiting the fungal cap RNA (guanine-N7) methyltransferase Abd1 but not the orthologous human cap methyltransferase enzyme RNMT/Hcm1 [4, 5].

The RNA (guanine-N7) methyltransferase Ecm1 from the microsporidian parasite *Encephalitozoon cuniculi* [6, 7], a 298-amino acid (aa) monomeric protein, suffices for cap methylation *in vivo*, as gauged by complementation in yeast [6]. Crystal structures of Ecm1 as binary complexes with SAM, SAH, and GTP delineated the binding pockets for the methyl donor and the methyl acceptor [8]. Comparisons of the substrate-bound Ecm1 structures suggested a direct in-line mechanism of methyl transfer without any direct enzymic contacts with the guanine-N7 nucleophile, the SAM methyl carbon, or the SAH sulfur leaving group, signifying that cap methylation occurs by optimizing proximity and geometry of the donor and acceptor [9], similar to the enzyme glycine N-methyltransferase [10]. Methylation of GTP by Ecm1 in the presence of 5 μM SAM was inhibited by the reaction product SAH (IC_50_ 4 μM) and by the substrate analog SFG (IC_50_ 1.5 μM) [11]. These findings indicate that (i) Ecm1 has similar affinity for the substrate SAM and the product SAH and (ii) SFG is a modest inhibitor of Ecm1 with only three-fold greater affinity for the methyl acceptor site than SAH. The structure of an Ecm1•SFG binary complex revealed a hydrogen bond from the SFG C-NH_2_ amine to a main-chain carbonyl [in lieu of the van der Waals (VDW) contact to the carbonyl made by the SAM methyl group] that would explain the differential affinity [7].

In contrast, the fungal *Saccharomyces cerevisiae* cap methyltransferase Abd1 (*Sc*Abd1) is exquisitely sensitive to inhibition by SFG, to which it binds up to 900-fold more tightly than to SAM or SAH [7]. However, due to lack of structural information, the atomic interactions underlying the inhibition of *Sc*Abd1 by SFG are poorly understood. *Sc*Abd1, a 436-aa monomeric enzyme, was the first cellular cap methyltransferase assigned to an essential cellular gene [12]. Extensive biochemical and genetic analyses of *Sc*Abd1 [12–15] showed that the N-terminal 109-aa segment is dispensable for methyltransferase activity *in vitro* and *in vivo* and identified the amino acids essential for cap methylation within the C-terminal catalytic domain [13]. It was inferred that Abd1 is the immediate target for SFG’s antifungal activity *in vivo* on the basis of the following evidence: (i) isogenic *S. cerevisiae* strains containing fungal versus mammalian mRNA capping systems [5] displayed five-fold differences in sensitivity to growth inhibition by SFG that correlated with the source of the cap (guanine-N7) methyltransferase component; (ii) the susceptibility of *S. cerevisiae* to growth inhibition by SFG was diminished when *Sc*Abd1 was overexpressed by increased gene dosage [7]; and (iii) increased gene dosage of yeast SAM synthase plus Abd1 afforded greater resistance to SFG than either enzyme alone [7].

In the present study, we employed biochemical, structural, and genetic means to define the basis for the distinctive SFG sensitivity of the fungal cap methyltransferase Abd1. Using a sensitive mass spectrometry (MS)-based assay of guanine-N7 methylation, we found that *Sc*Abd1 and its *Kluyveromyces lactis* ortholog *Kl*Abd1 are, respectively, 485-fold and 245-fold more sensitive to inhibition by SFG than by SAH. We determined crystal structures of *Kl*Abd1 and *Sc*Abd1 ternary complexes with GTP and SFG that revealed two key new contacts between SFG and GTP compared to the respective Abd1•SFG binary complexes, comprising (i) a hydrogen bond from the SFG C-NH_2_ amine to the guanine-O6 atom and (ii) a hydrogen bond from the SFG C-NH_2_ amine to the guanine-N7 atom. These additional interactions rationalize the higher potency of SFG versus SAH against the fungal RNA (guanine-N7) methyltransferases.

To fortify the case for Abd1 as the target of SFG antifungal efficacy, we isolated three SFG-resistant *S. cerevisiae* strains with two or more missense mutations in Abd1, the common thread being a substitution at Tyr416 (by Cys, Phe, or Asn). We demonstrated that a single alanine substitution for Tyr416 sufficed to confer SFG resistance *in vivo* and that each of the *Sc*Abd1 variants displayed reduced sensitivity to inhibition by SFG *in vitro*. The crystal structure of an SFG-resistant *Sc*Abd1 variant (K163R-K311R-F387Y-Y416F) revealed that whereas the contacts between SFG and the cap guanosine are maintained, the methyl acceptor site is perturbed due the Y416F substitution. Taken together, our results provide insights into the sensitivity and acquired resistance of fungal RNA guanine-N7 methyltransferases to SFG.

## Materials and methods

### Production of recombinant fungal cap methyltransferases in bacteria

DNAs encoding full-length *K. lactis* (*Kl*) Abd1 (UniProt accession Q6CKI0; aa 1–426) and N-terminal truncation variants *Kl*Abd1-(117–426) (Δ116) and *Kl*Abd1-(138–426) (Δ137), and full-length *S. cerevisiae* (*Sc*) Abd1 (UniProt accession A6ZLH5; aa 1–436) and N-terminal truncation variants *Sc*Abd1-(120–436) (Δ119) and *Sc*Abd1-(141–436) (Δ140) were inserted into pSMT3 so as to fuse the genes in-frame with a leader sequence encoding an N-terminal His_10_-Smt3 tag [16]. *Sc*Abd1 was also inserted in-frame into pET16b-His_10_ using NdeI and BamHI restriction sites. Missense mutations were introduced into the *Sc*Abd1 expression plasmids by polymerase chain reaction (PCR) with a QuickChange kit (Stratagene). The inserted DNA sequences were sequenced completely to exclude the acquisition of unwanted coding changes during DNA amplification and cloning. The *Kl*Abd1 and *Sc*Abd1 plasmids were transformed into *Escherichia coli* BL21 (DE3) CodonPlus RIL (Novagen). For expression of *Kl*Abd1 and *Sc*Abd1, 4-l bacterial cultures, supplemented with 50 μg/ml kanamycin (pSMT3) or 100 μg/ml carbenicillin (for pET16b-*Sc*Abd1 wild-type and SFG-resistant variants), 100 μg/ml chloramphenicol, and 100 μl/l antifoam 204 (Sigma, USA), were grown at 37°C in baffled flasks to an *A*_600_ of 2. The temperature was then reduced to 20°C, isopropyl-β-d-thiogalactoside was added to 0.5 mM, and the cultures were incubated overnight at 20°C.

### Cap methyltransferase purification

Bacteria were harvested by centrifugation at 7000 × *g* and suspended in buffer containing 20 mM HEPES (pH 7.5), 500 mM NaCl, 20 mM imidazole, 0.1% IGEPAL, 20% sucrose, 0.02% NaN_3_, 1 mM β-mercaptoethanol (βME), 5 μg/ml DNase, and 10 μg/ml lysozyme. Cells were disrupted by sonication and debris was removed by centrifugation at 45 000 × *g*. The supernatants were applied to a chromatography column packed with 10 ml (or 5 ml for small-scale preparations) of His_60_ superflow resin (Clontech, USA) that had been equilibrated with buffer A (20 mM HEPES, pH 7.5, 20 mM imidazole, 500 mM NaCl, 1 mM βME). The columns were washed with buffer A and the His_10_-Smt3 or His_10_ tagged proteins were eluted with buffer B (20 mM HEPES, pH 7.5, 350 mM NaCl, 250 mM imidazole, 1 mM βME). The N-terminal His_10_-Smt3 tag was cleaved by overnight digestion at 4°C with the Smt3-specific protease Ulp1 [16] at a 2000:1 ratio of RNA cap methyltransferase:Ulp1. The tag-free methyltransferases were then separated from the tag by gel filtration through a Superdex 200 (16/60) column equilibrated with buffer containing 20 mM HEPES, pH 7.5, 350 mM NaCl, and 1 mM βME. The elution profiles were monitored by *A*_280_ and sodium dodecyl sulfate–polyacrylamide gel electrophoresis. For crystallization experiments, the peak Abd1 fractions were pooled and further purified by adsorption to an anion exchange column (Mono Q 10/100) and elution with a linear 0.2–1 M NaCl gradient. Purified Abd1 proteins were concentrated to 15–20 mg/ml (Supplementary Fig. S1) and buffer-exchanged to 20 mM HEPES (pH 7.5), 150 mM NaCl, and 5 mM βME using a 10-kDa Amicon Ultra-15 centrifugal filter device (Millipore, USA), and then flash frozen in liquid nitrogen and stored at −80°C.

### Analytical size exclusion chromatography

A Superdex S200 (10/300) column equilibrated in 20 mM HEPES (pH 7.5), 150 mM NaCl, and 5 mM βME was used to analyze the native sizes of the purified cap methyltransferases. The column was calibrated with Gel Filtration Standards (Bio-Rad; catalog #151-1901) composed of thyroglobulin (MW 670 kDa), bovine γ-globulin (MW 158 kDa), chicken ovalbumin (MW 44 kDa), horse myoglobin (MW 17 kDa), and vitamin B12 (MW 1.35 kDa). The column flow was set to 0.75 ml/min.

### Cap methyltransferase substrates, products, and inhibitors

Solutions of SAM (Cayman Chemical), SAH (Millipore-Sigma), SFG (Santa Cruz Biotechnology), GTP (Jena Bioscience), m^7^GTP (Millipore-Sigma), GpppA (Jena Bioscience), and m^7^GpppA (Jena Bioscience) were prepared in 300 mM HEPES buffer (pH 7.5), and concentrations were determined from the absorbance at 260 nm using the following extinction coefficients (M^−1^ cm^−1^): SAM, 16000; SAH, 15400; SFG, 16000; GTP, 13700; m^7^GTP, 9800; GpppA, 27000; and m^7^GpppA, 22700.

### Assay of cap methyltransferase activity

**Figure 1.**
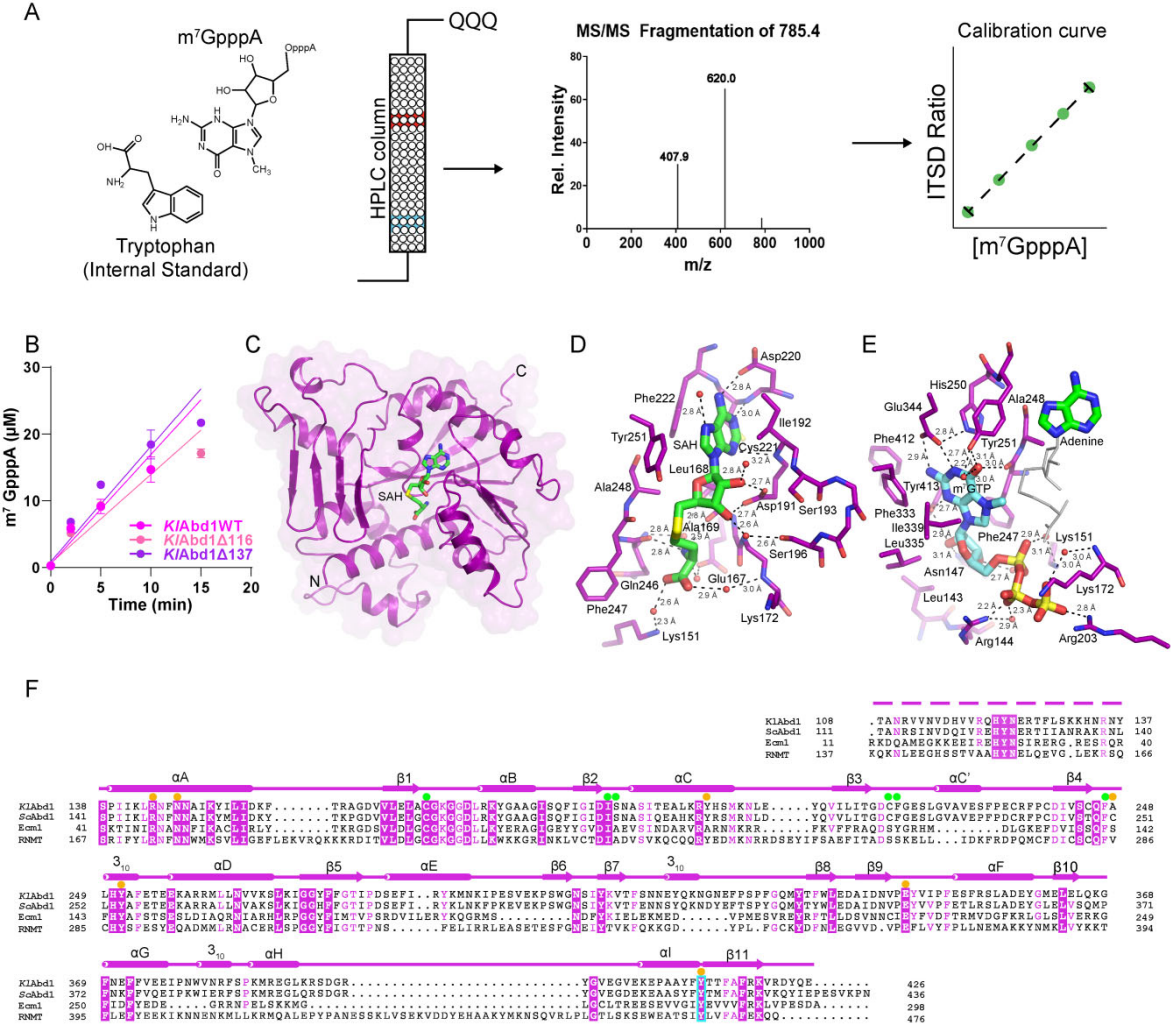
Structures of *K. lactis* RNA cap methyltransferase in complexes with products SAH and m^7^GTP. (**A**) Schmatic outline of the experimental setup for methylation assays. Methylation of substrate (m^7^GpppA) was detected by separating reactions products (in red and blue) using a high-performance liquid chromatography (HPLC) column coupled to a triple quadrupole (QQQ) MS, which detects product ions (see the “Materials and methods” section). Quantification was achieved by normalizing ion counts to an internal standard, l-tryptophan. The ratio of m^7^GpppA ion counts to l-tryptophan ion counts yielded the internal standard ratio (ISTD). A range of m^7^GpppA calibration standards (with constantl-tryptophan) provided a linear calibration curve between ISTD and m^7^GpppA concentration. Sample ISTDs were then used to interpolate a discrete m^7^GpppA concentration from the calibration curve. (**B**) Methylation activities of wild-type (purple circles) and two truncated *Kl*Abd1 variants (*Kl*Abd1Δ116, pink circles; and *Kl*Abd1Δ137, dark pink circles) with GpppA substrate (see the “Materials and methods” section). Error bars (one standard deviation) are calculated from three independent experiments performed in triplicate. (**C**) A view of the *Kl*Abd1 structure (colored purple) as a ribbon with arrows for β-strands and wide ribbons for helices. A transparent molecular surface envelops the structure. N and C denote amino and carboxyl termini, respectively. Bound SAH in the methyl donor site is shown in stick representation (green). (**D**) A closeup view of the methyl donor site of *Kl*Abd1–SAH complex. (**E**) A view of the methyl acceptor site of *Kl*Abd1 bound to m^7^GTP (stick representation in cyan). Bound adenine in the methyl donor site is shown in stick representation (green) and rest of the unresolved substrate/product is depicted in thin gray line. Side chains shown in stick representation, and waters are denoted by red spheres. Atomic contacts are indicated by dashed lines with distances. (**F**) Aligned primary structures of cap methyltransferases from *K. lactis* (*Kl*Abd1), *S. cerevisiae* (*Sc*Abd1), *E. cuniculi* (Ecm1), and *Homo sapiens* (RNMT). The secondary structure elements of *Kl*Abd1 are shown above the amino acid sequences, with α-helices depicted as cylinders and β-strand as arrows. Gaps in the alignments are indicated by “•”. Side-chain identity/similarity is denoted by shading and letter color (purple shade conserved in all; purple letters conserved in most). A predicted disordered region N-terminal to the catalytic domain is denoted by a purple dashed line above the alignment. *Kl*Abd1 amino acids that contact SAH and m^7^GTP are indicated by green and orange circles, respectively. A conserved tyrosine in β11 that interacts with the cap guanosine is outlined in cyan.

Reactions (50 μl) were performed in triplicate by incubating 250 nM cap methyltransferases with 100 μM SAM and 250 μM GpppA or 2 mM GTP at 30°C in a buffer consisting of 200 mM NaCl, 50 mM Tris–HCl (pH 8.2), and 5 mM βME. Samples (10 μl aliquots) were withdrawn at specified time points (*Kl*Abd1s: 0, 2, 5, 10, and 15 min, Fig. 1A; *Sc*Abd1s: 0, 5, 10, 15, and 20 min, Fig. 4A) and quenched with 10 μl of 100 mM H_2_SO_4_ and 50 μM l-tryptophan, and then stored at −80°C pending liquid chromatography-tandem mass spectrometry (LC–MS/MS) analyses.

### LC–MS/MS analysis

The quenched samples were thawed and centrifuged at 4000 × *g* at 4°C for 30 min to pellet denatured protein and the supernatants were transferred to a 96-well plate for analysis. Methylation was measured via HPLC with detection by MS conducted on an Agilent 1200 HPLC system coupled with a 6490 QQQ (Agilent Technologies). Assay samples were separated on an InfinityLab Poroshell 120 HILIC-Z column (2.1 mm × 100 mm, 2.7 μm particle size; Agilent Technologies) equilibrated in NH_4_Ac buffer [A: 10% 10 mM NH_4_Ac, pH 9.0, and 500 μM methylene diphosphonic acid/90% acetonitrile (ACN); B: 90% 10 mM NH_4_Ac, pH 9.0, and 500 μM methylene diphosphonic acid/10% ACN] with a flow rate 400 μl/min. The eluents of the column were directed to an electrospray ion source, connected to the QQQ. Samples were analyzed by the QQQ using multiple reaction monitoring (MRM) in negative mode, which measures peak intensities of the product ions of m^7^GpppA (785: 408, 620 *m*/*z*) or m^7^GTP (536.0: 159.0, 371.0 *m*/*z*) and the internal standard, l-tryptophan (203: 116, 141.9 *m*/*z*). The resultant peak intensities were then integrated. l-Tryptophan was used as an internal standard to perform cross-sample analysis. The m^7^GpppA or m^7^GTP product ion integrated intensities were normalized to the integrated ion intensity of l-tryptophan, yielding the ISTD. A range of m^7^GpppA concentrations (0, 0.5, 1, 2, 4, 8, 16, 32, and 64 μM) and m^7^GTP concentrations (0, 0.3, 0.9, 8.1, 24.3, and 72.9 μM) with a constant l-tryptophan concentration (50 μM) generated a linear calibration curve when respective ISTDs were plotted as a function of m^7^GpppA or m^7^GTP concentrations. ISTDs obtained from reaction samples quenched at specified time points were then used to interpolate either m^7^GpppA or m^7^GTP concentrations using the calibration curves and used for quantification. Prior to running the MRM protocol and subsequent analyses, the QQQ was tuned using Calibration Standard Tuning Mix (Agilent Technologies). Data collection and integration of peak intensities were carried out by the MassHunter software package.

### Inhibition of methyltransferase activity by SAH or SFG

The inhibitory effects of SAH and SFG on *Kl*Abd1 and *Sc*Abd1 (250 nM) were assayed using the GpppA methylation assay conditions described above. Reaction mixtures (45 μl) with cap methyltransferases were incubated with increasing concentrations of SAH (0, 3.125, 12.5, 25, 50, 100, 200, and 800 μM) or SFG (0, 15.6, 31.3, 62.5, 125, 250, and 500 nM) for 10 min prior to the addition of the GpppA methyl acceptor. Reactions were quenched after incubation at 30°C for 10 min (for *Kl*Abd1) or 20 min (for *Sc*Abd1). *Sc*Abd1 SFG-resistant variants were tested with a higher range of SFG concentrations: 0, 0.10, 0.50, 0.75, 1.2, 1.7, 2.2, and 4 μM SFG.

### Statistical analysis

All analyses were conducted in R (version 4.3.1) [17]. Dose response analyses were performed using a four-parameter log-logistic regression model using the *drc* package [18]. Visualization of analysis was created by the ggplot2 package [19].

### Protein crystallization

Crystallization trials of the fungal cap methyltransferases (wild-type and variants) were performed by sitting drop vapor diffusion by mixing 400 nl of protein solution (15–20 mg/ml) with an equal volume of precipitant solution—from MCSG (Microlytic), Index HT, Crystal Screen HT, and Peg Ion HT (Hampton Research) sparse matrix crystallization suites—using a Crystal Gryphon (Art Robbins Instruments). Initial crystal “hits” were further optimized with several rounds of grid screening using a Formulator (Art Robbins Instruments). Co-crystallized ligands, optimized crystallization conditions, and cryo-protectants used for crystal freezing are summarized in Supplementary Table S1.

### Data collection and processing, structure determination, model building, refinement, and analysis

Diffraction data from single crystals were collected with a CCD ADSC QUANTUM 315 (BNL X29A), Pixel Dectris Eiger 9M (BNL AMX ID-17-1), or Rayonix MX-225 HE detector (APS LRL-CAT ID-31) (Supplementary Table S1). Data were integrated and scaled using either HKL-3000 or AIMLESS [20]. Initial phases were determined by molecular replacement using Phaser [20] and atomic models were built into the respective densities using COOT [21]. The model was iteratively adjusted and refined with REFMAC5 [20, 22]. The refined model of *Kl*Abd1Δ137 was used as a search model for subsequent structure determinations of the other fungal methyltransferases. These structures were refined with either REFMAC5 or Phenix [23]. Analyses of the structures were performed in COOT and MOLPROBITY [24]. Crystallographic data and refinement statistics and RCSB accession codes are compiled in Supplementary Table S1. The structural models exhibited excellent geometry with no residues in disallowed regions of the Ramachandran plot [25].

### Isolation of SFG-resistant *S. cerevisiae* with mutations in the *ABD1* gene

A library of PCR-mutated *ABD1* alleles (ABD1*) in a *CEN TRP1* plasmid under the control of the *ABD1* promoter was constructed as described [14]. The pABD1* library DNA was transfected into *S. cerevisiae* strain *abd1*Δ pABD1 (*CEN URA3 ABD1*) pSAM3 (*2µ HIS3 SAM3*) [26]. Trp^+^ His^+^ transformants were selected and then plated to agar medium containing 5-fluoroorotic acid (FOA) to counter-select against the *CEN URA3 ABD1* plasmid. FOA survivors were plated to Trp- His- agar plates (25 ml) to which 300 µl of a 2.5 or 5 µM solution of SFG had been applied. Colonies that grew well on SFG-containing plates were candidates for harboring SFG-resistant ABD1* alleles. To ascertain whether SFG-resistance was linked to *ABD1*, the plasmids were recovered from individual SFG-resistant strains and amplified clonally by transformation in *E. coli*. The pABD1* plasmid DNAs isolated from *E. coli* were transfected into *S. cerevisiae abd1*Δ pABD1 (*CEN URA3 ABD1*) pSAM3 (*2µ HIS3 SAM3*) strain. Trp^+^ His^+^ transformants were selected and plated to FOA for counterselection against the *CEN URA3 ABD1* plasmid. The resulting *abd1*Δ pABD1* (CEN TRP1 ABD1*) pSAM3 (*2µ HIS3 SAM3*) strains were tested in parallel with a control *abd1*Δ pABD1 (*CEN TRP1 ABD1*) pSAM3 (*2µ HIS3 SAM3*) strain for growth on Trp- His- agar plates (25 ml) supplemented with SFG (300 µl of 0.63, 1.25, 2.5 and 5 µM SFG per plate). Three of the ABD1* strains were less sensitive to growth inhibition by SFG than the isogenic wild-type *ABD1* strain, signifying that SFG resistance was linked to the pABD1* plasmid. These pABD1* plasmids were sequenced to detect any coding changes within the *ABD1* gene.

## Results

### Methyltransferase activity of *K. lactis* Abd1

Full-length *K. lactis* cap methyltransferase *Kl*Abd1 (426-aa) and two N-terminal deletion variants—*Kl*Abd1Δ116 and *Kl*Abd1Δ137—were produced in *E. coli* and purified. The recombinant enzymes eluted as monomers on size exclusion chromatography (Supplementary Fig. S1A and B). To gauge enzymatic activity, we implemented a sensitive MS-based assay (Fig. 1A). Methylation of substrates GpppA or GTP was detected by separating reactants and products m^7^GpppA or m^7^GTP using an HPLC column coupled to a QQQ MS, which detects product ions. Quantification was achieved by normalizing ion counts to an internal l-tryptophan standard. The ratio of m^7^GpppA or m^7^GTP ion counts to l-tryptophan ion counts yielded the ISTD. A linear calibration curve was generated by plotting ISTDs obtained from a range of m^7^GpppA (or m^7^GTP) concentrations with a constant l-tryptophan concentration as a function of respective m^7^GpppA (or m^7^GTP) concentration (Fig. 1A). ISTDs of reaction sampled at 0, 2, 5, 10, and 15 min were then used to interpolate m^7^GpppA (or m^7^GTP) concentration from the calibration curve, and product formation was plotted as a function of time (Fig. 1B). From the slope of the linear fit, we estimated that the turnover rate of wild-type *Kl*Abd1 for GpppA was 5.4 ± 0.5 min^−1^. The reaction rates of the *Kl*Abd1Δ116 and *Kl*Abd1Δ137 variants (5.3 ± 0.4 and 6.9 ± 0.6 min^−1^, respectively) were similar to that of the full-length enzyme (Fig. 1B).

### Structure and active site of *K. lactis* Abd1

A crystal of *Kl*Abd1Δ137 that had been preincubated with 0.7 mM SAH diffracted X-rays to 1.3 Å resolution, was in space group *P*2_1_ and contained two enzyme protomers in the asymmetric unit. The structure was determined by molecular replacement in Phaser by using *E. cuniculi* cap methyltransferase Ecm1 (PDB 2HV9) [7] as a search model. Clear electron densities for SAH were observed in the methyl donor site of both Abd1 protomers (Fig. 1C and D, and Supplementary Fig. S3A). The final model was refined at 1.3 Å resolution with *R*_work_/*R*_free_ of 16.5/18.7 (Supplementary Table S1). The two protomers in the asymmetric unit are nearly identical and are superimposable with an root mean square deviation (r.m.s.d) of 0.4 Å over 289 Cα positions. Subsequent depictions and discussions of the *Kl*Abd1Δ137–SAH complex will refer to protomer A.

*Kl*Abd1Δ137 contains structural motifs that are characteristic of the class 1 methyltransferases [27, 28], which include a seven-stranded (β1–β5 and β10–β11) central β-sheet surrounded by helices αA–αD, helix αF, and one 3_10_ helix. In addition, *Kl*Abd1Δ137 contains RNA cap methyltransferase-specific structural elements: (i) an α/β domain (aa 281–346) composed of a four-stranded (β6–β9) anti-parallel β-sheet, helix αE, and a 3_10_ helix; and (ii) a C-terminal three helix cluster (helices αG–αI) and a 3_10_ helix, together spanning aa 368–411 (Fig. 1F).

### Structural comparison of *Kl*Abd1Δ137 to Ecm1 and human RNMT

*Kl*Abd1 shares 32% sequence identity with the microsporidian parasite *E. cuniculi* cap methyltransferase, Ecm1 (Fig. 1F). The *Kl*Abd1Δ137 and Ecm1 structures superimpose with an r.m.s.d. of 1.2 Å over 241 Cα positions (Supplementary Fig. S2A). However, there are distinctions between the two cap methyltransferases whereby (i) the segment between β7 and β8 (aa 310–331) of *Kl*Abd1Δ137 contains an insertion that forms a 3_10_ helix and an extended loop and buttresses the class 1 methyltransferase domain and (ii) the segment between helices αG and αI (aa 375–405) of *Kl*Abd1 forms an additional 3_10_ helix and distinctly reorganizes the tertiary structure of the cap methyltransferase-specific α/β domain.

The structure of the catalytic domain of human cap methyltransferase RNMT, which shares 36% sequence identity with *Kl*Abd1 (Fig. 1F), has been determined in the absence and presence of its activating partner RAM (RNMT activating miniprotein) [29]. Structure-based sequence alignment highlights that RNMT contains an insertion (residues 416–456) between αG and αI that is not present in *Kl*Abd1 (Fig. 1F). In the RAM-free structure of RNMT (PDB 3BGV), the insertion is disordered; hence, the overall structure is similar to *Kl*Abd1 and is superimposable with an r.m.s.d. of 1.6 Å over 249 Cα positions (Supplementary Fig. S2B). In RAM-bound RNMT (PDB 5E8J), this region adopts an RNMT-specific fold (an RNMT lobe) comprising two β-strands (β10a and β10b) and an α-helix (αL) and interacts with the RAM (Supplementary Fig. S2C) [29].

### Methyl donor site of *Kl*Abd1Δ137 occupied by product SAH

SAH is a product of SAM-dependent methylation and a competitive inhibitor of cap methylation reactions [7]. In the *Kl*Abd1 crystal structure, SAH occupies the methyl donor site in a conformation similar to that observed previously for SAM, SAH, and aza-SAM in Ecm1 or RNMT [9, 11 ,29] and SAH interacts with an ensemble of conserved residues in a manner similar to Ecm1 and RNMT [9, 29]. The adenine base of SAH is sandwiched between the side chains of Ile192 and Phe222 and is partially shielded from the solvent by the side chain of Tyr251 (Fig. 1D). Adenine-N1 and -N6 form hydrogen bonds with the Cys221 amide nitrogen and Asp220-Oδ1, respectively. Adenine-N3 engages in a hydrogen bond with the Ile192 amide nitrogen. The ribose O2′ and O3′ atoms form a bidentate hydrogen bond with Asp191-Oδ1 and Oδ2 (Fig. 1D). The ribose O3′ makes additional water-mediated contact to Ser196-Oγ. The ribose O4′ contacts Ala169 and Ala248 through VDW interactions. The SAH amino nitrogen donates hydrogen bonds to the main-chain carbonyl oxygens of Ala169 and Gln246 and makes water-mediated contacts with Glu-Oε2, the Leu168 and Ala169 main-chain carbonyls, and the Gln246 main-chain amide. The SAH carboxylate oxygens are engaged in water-mediated interactions with Lys151-Nζ and the Lys172 main-chain amide (Fig. 1D).

### Methyl acceptor site of *Kl*Abd1Δ137 occupied by m^7^GTP product

RNA cap methyltransferases accept GTP as a substrate and produce m^7^GTP [7, 30], which is a mimetic of the methylated cap. Here we co-crystallized *Kl*Abd1Δ137 with 0.9 mM m^7^GTP and determined the structure using *Kl*Abd1Δ137-SAH as a search model. The final model was refined at 1.33 Å resolution with *R*_work_/*R*_free_ of 18.5/20.3 (Supplementary Table S1). The asymmetric unit comprised two *Kl*Abd1 protomers. Electron densities for m^7^GTP were observed in both methyl acceptor sites (Fig. 1E and Supplementary Fig. S3B). Additional electron densities were observed in the methyl donor sites, which were interpreted as the co-purified adenine moiety of the product SAH (Supplementary Fig. S3B). The tertiary structure of m^7^GTP-bound *Kl*Abd1Δ137 was virtually identical to that of SAH-bound *Kl*Abd1Δ137 (r.m.s.d. of 0.4 Å over 287 Cα positions).

The guanine moiety of m^7^GTP is buried in a deep pocket created by Phe247, Ala248, His250, Phe333, Ile339, and Phe412 (Fig. 1E). The guanine-N1 and -N2 atoms make a bidentate hydrogen bond with the Glu344-Oε1 and Oε2. Guanine-O6 makes direct and water-mediated hydrogen bonds with Tyr251-OH. Guanine-N3 makes a hydrogen bond to Tyr413-OH. The guanosine of m^7^GTP adopts an *anti* conformation, as was observed in the cap-bound Ecm1 complex [9]. The ribose ring is flanked by Phe247 and Leu335, with which it makes VDW interactions (Fig. 1E). The ribose O2′ makes hydrogen bonds with Asn147-Nδ2 and Tyr413-OH. The ribose O3′ makes a hydrogen bond with Asn147-Nδ2. The triphosphate of m^7^GTP is surrounded by four basic residues: Arg144, Lys151, Lys172, and Arg203 (Fig. 1E). The γ-phosphate makes a direct contact with Arg203-NH1 and a water-mediated interaction with the Lys172 main-chain amide. The β-phosphate makes direct and water-mediated interactions with Arg144-NH1. The α-phosphate makes a water-mediated interaction with Lys151-Nζ. Additionally, the α-phosphate interacts with the ribose O3′ via a water.

### Potent inhibition of *Kl*Abd1 by SFG

SFG is a natural product SAM analog in which the S-CH_3_ sulfonium moiety of SAM is replaced by a C-NH_2_ amine. SFG has antiprotozoal, antifungal [31], and antiviral activities [32–34]. SFG inhibits the growth of *S. cerevisiae* and an *S. cerevisiae* strain complemented with *Candida albicans* capping enzymes with equal potency [4, 35]. SFG is five-fold less potent against an *S. cerevisiae* strain complemented with the mammalian capping apparatus [5]. The observations, and the finding that overexpression of Abd1 in *S. cerevisiae* confers resistance to SFG [2], argue that cap methyltransferase is the target of SFG inhibition of yeast growth. SFG exhibited a concentration-dependent inhibition of *S. cerevisiae* Abd1 *in vitro*, with an apparent IC_50_ of 24 nM [7]. This suggests that the preferential inhibition of yeast cell growth expressing fungal cap methyltransferases might stem from the natural susceptibility of these fungal enzymes to SFG.

**Figure 2.**
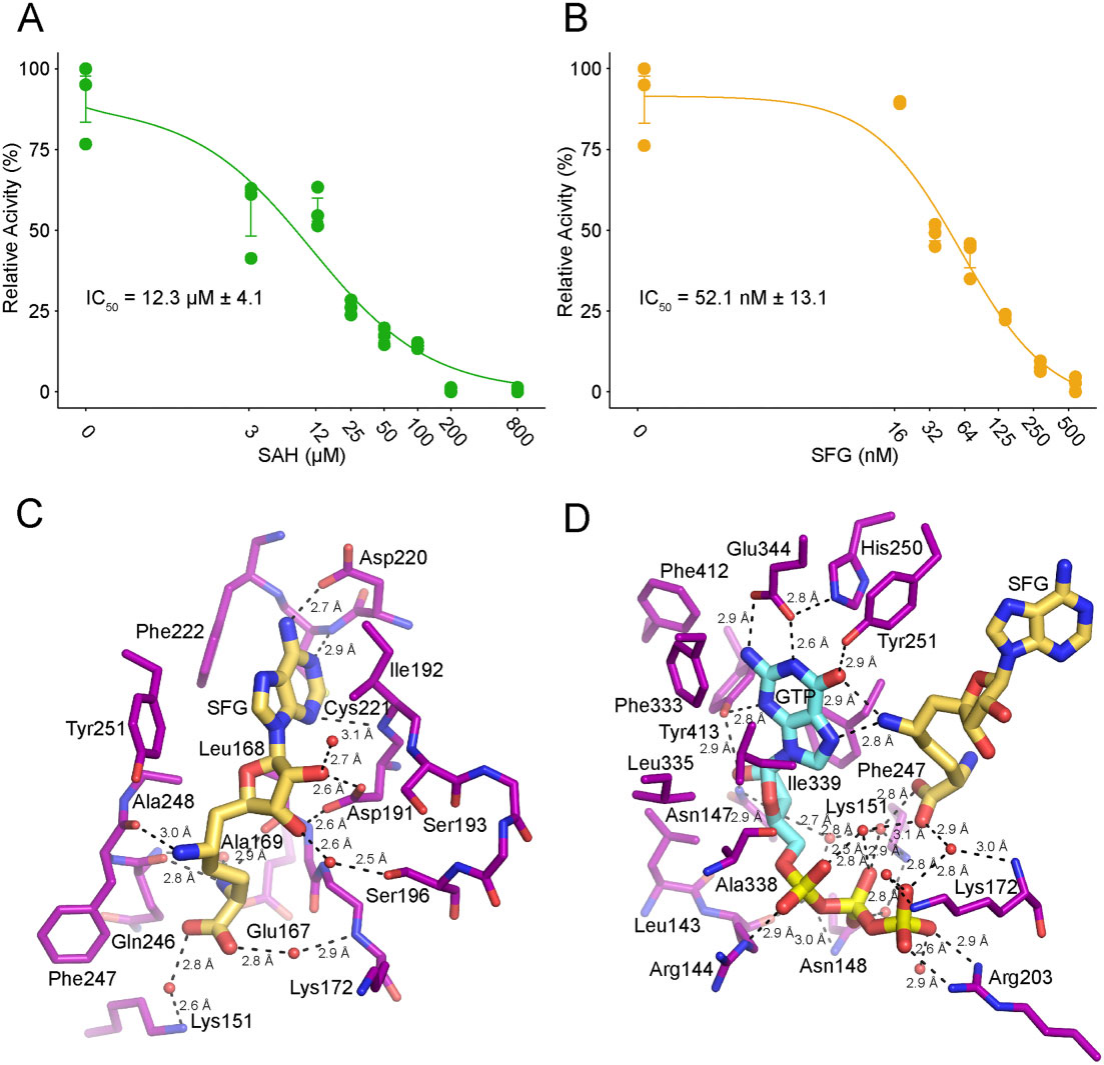
Structure of *K. lactis* Abd1 bound to inhibitor SFG and SFG plus GTP. (**A**) SAH inhibition. The extent of methylation of GpppA by wild-type *Kl*Abd1 was quantified (see the “Materials and methods” section) in the presence of increasing concentrations (0, 3.125, 12.5, 25, 50, 100, 200, and 800 μM) of SAH and the obtained relative activities (%) for the enzyme (green circles) were plotted as a function SAH concentrations to determine the IC_50_ value (indicated). (**B**) SFG inhibition. The extent of methylation of GpppA by wild-type *Kl*Abd1 was quantified (see the “Materials and methods” section) in the presence of increasing concentrations (0, 15.6, 32.25, 62.5, 125, 250, and 500 nM) of SFG and the relative activities (%) for *Kl*Abd1 (light yellow circles) were plotted as a function SFG concentrations to determine the IC_50_ value (indicated). Error bars (one standard deviation) are calculated from three independent experiments performed in triplicate. (**C**) A view of the methyl donor site of *Kl*Abd1 bound to SFG shown in stick representation and colored light yellow. (**D**) A closeup view of the methyl acceptor site of *Kl*Abd1 bound to SFG (as in panel B) and GTP (stick representation in cyan). Side chains are shown in stick representation (as in Fig. 1), and waters are denoted by red spheres. Atomic contacts are indicated by dashed lines with distances.

Here we find that SFG inhibits *Kl*Abd1 *in vitro* with an apparent IC_50_ of 52.1 ± 13.1 nM (Fig. 2B). In contrast, the product SAH inhibits *Kl*Abd1 with an IC_50_ of 12.3 ± 4.1 μM (Fig. 2A). From the ratio of the IC_50_ values, we conclude that SFG is 240-fold more potent than SAH in inhibiting fungal cap methylation.

### Structure of *Kl*Abd1Δ137 in complex with SFG

To interrogate the basis for SFG’s high potency vis-à-vis SAH, we co-crystallized *Kl*Abd1Δ137 with 0.6 mM SFG. The structure was determined by molecular replacement and the final model was refined at 1.59 Å resolution with *R*_work_/*R*_free_ of 18.7/21.9 (Supplementary Table S1). The atomic interactions of *Kl*Abd1 with SFG (Fig. 2C) recapitulate the interactions observed in the SAH complex (Fig. 1C), with a few key exceptions, as follows: (i) an additional contact between the Phe247 backbone carbonyl and the C-NH_2_ amine of SFG; (ii) closer contact distance between Asp220-Oδ1 with adenine-N6 (SFG, 2.7 Å versus SAH, 2.8 Å); (iii) closer contacts between Cys221 (N) and N1 positions of adenine (SFG, 2.9 Å versus SAH, 3.0 Å); (iv) closer contacts between Asp191 (Oδ1) and the ribose-O2′ (SFG, 2.6 Å versus SAH, 2.7 Å); and (v) closer contact of Asp191 (Oδ1) to the ribose-O3′ (SFG, 2.6 Å versus SAH, 2.7 Å).

### Ternary complex of *Kl*Abd1Δ137, SFG, and GTP

We proceeded to co-crystallize *Kl*Abd1Δ137 with 0.6 mM SFG and 1 mM GTP and determined the structure at 1.42 Å resolution with *R*_work_/*R*_free_ of 17.4/20.1 (Supplementary Table S1). Electron densities for SFG and GTP were evident in the methyl donor and acceptor sites of both protomers (Supplementary Fig. S3D). All of the atomic contacts to SFG in the binary complex are retained in the ternary complex (Fig. 2C and D). However, the structure of the ternary complex reveals key new contacts compared to the SFG binary complex, i.e. hydrogen bonds from the SFG C-NH_2_ amine to the guanine-O6 (2.9 Å) and N7 (2.8 Å) atoms of GTP (Fig. 2D). These two additional hydrogen bonding interactions specific to SFG might well account for the 240-fold higher potency of SFG versus SAH for *Kl*Abd1 (Fig. 2A and B).

In the ternary complex, GTP is bound in the methyl acceptor site (Fig. 2D) in a conformation analogous to the m^7^GTP of the *Kl*Abd1Δ137•m^7^GTP binary complex (Fig. 1E). All of the atomic contacts to m^7^GTP in the binary complex apply to GTP in the ternary complex (Fig. 2D). However, there is a change in the torsional angles around the α-phosphate when comparing the conformation of the bound m^7^GTP and GTP in the binary and ternary complexes, respectively. The torsional angle of the C5′–O5′ bond shows a change of −21°, going from −145° in m^7^GTP to −166° in GTP. As a consequence, the GTP α-phosphate makes a new and direct interaction with Arg144-Nϵ (2.9 Å) and an additional water-mediated contact with the Ala338 main-chain carbonyl. The α- and β-phosphates are engaged in water-mediated interactions with each other and with the SFG carboxylate. In addition, the β-phosphate makes new water-mediated interactions with the Arg144 main-chain carbonyl, with Asn148-Oδ1 and -Nδ2, and with Lys151-Nζ. The GTP γ-phosphate makes two additional water-mediated interactions compared to m^7^GTP: with Lys172-Nζ and the SFG carboxylate. We surmise that these conformational changes in the methyl acceptor are induced by the SFG carboxylate in the ternary complex.

### *In vivo* selection for SFG-resistant *S. cerevisiae* Abd1 variants

SFG (IC_50_ 24 nM) is 900-fold more potent than SAH (IC_50_ 21 μM) in inhibiting methylation of GpppA by recombinant *S. cerevisiae* Abd1 [7]. *Saccharomyces cerevisiae* was less sensitive to growth inhibition by SFG when *Sc*Abd1 was overexpressed. Overexpressing yeast SAM synthase plus *Sc*Abd1 afforded greater resistance to SFG than overexpressing either enzyme alone [26]. These results are consistent with the proposal that mRNA cap methylation is a principal target of SFG bioactivity. An initial attempt to identify missense mutations in *Sc*Abd1 that confer SFG resistance *in vivo* entailed selection for outgrowth of SFG-resistant cells from a pool of yeasts containing random PCR-mutagenized (but functional) ABD1* genes on a single-copy plasmid. Although forced passage in liquid culture in the presence of 10 μM SFG yielded resistant strains, the resistance determinants did not reside on the pABD1* plasmid. Genetic tests assigned SFG resistance to *SAM3* [26], which encodes the yeast high-affinity SAM transporter [36, 37]. Spontaneous SFG-resistant Sam3 mutations resulted in loss of function in SAM uptake, as gauged by growth on medium containing exogenous SAM as the sulfur source. Overexpression of wild-type Sam3 increased the sensitivity of yeast to growth inhibition by SFG. These results highlighted SFG uptake by a SAM transporter as a critical determinant of its antifungal activity.

**Figure 3.**
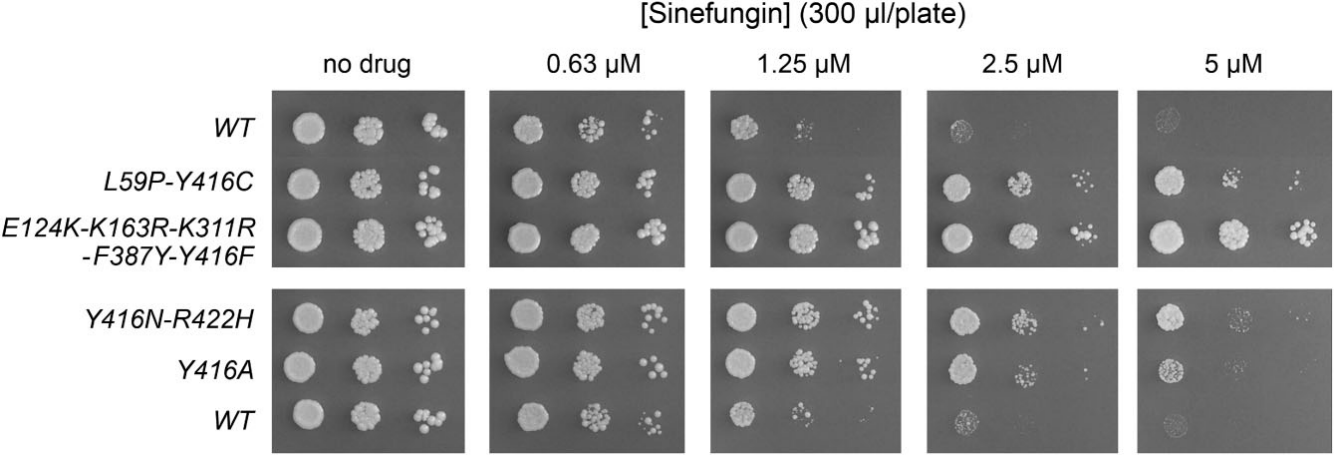
SFG-resistant *S. cerevisiae* Abd1 variants. Serial 10-fold dilutions of *abd1*Δ pSAM3 (*2μ HIS3 SAM3*) strains bearing *ABD1* alleles (as specified on the left) on *CEN TRP1* plasmids were spot-tested for growth at 30°C on Trp^-^ His^-^ agar (25 ml per plate) supplemented with SFG as indicated at the top.

Here we revisited the effort to isolate SFG-resistant *Sc*Abd1 variants by transforming the mutagenized p(CEN TRP1 ABD1*) plasmid library into an *abd1*Δ p(*CEN URA3 ABD1*) strain in which the *SAM3* gene is placed on a multicopy *2μ HIS3* plasmid, thereby making it unlikely that an inactivating mutation in the chromosomal *SAM3* gene, or any of the plasmid-borne genes, will result in SFG resistance in the presence of an excess of wild-type *SAM3* alleles. After plasmid shuffle to exchange the ABD1* alleles for wild-type *ABD1*, we screened for individual colonies that grew on agar medium containing SFG. The pABD1* (CEN TRP1 ABD1*) plasmid was isolated from individual SFG-resistant colonies, amplified in *E. coli* and introduced into the *abd1*Δ pABD1 (*CEN URA3 ABD1*) pSAM3 (*2μ HIS3 SAM3*) strain by plasmid shuffle. The resulting *abd1*Δ pABD1* pSAM3 strains were retested for SFG sensitivity by spotting serial dilutions of cell suspensions to agar plates supplemented with increasing concentrations of SFG. Three of the ABD1* strains were less sensitive to growth inhibition by SFG than the isogenic wild-type *ABD1* strain, signifying that SFG resistance was linked to the plasmid (Fig. 3). The pABD1* plasmids from these three strains were sequenced in order to detect any coding changes within the *ABD1* gene. Two of the alleles were double variants: L59P-Y416C and Y416N-R422H, respectively. The third allele had five missense changes: E124K-K163R-K311R-F387Y-Y416F. Note that all three alleles included a substitution for Tyr416, which implicates this amino acid as a key determinant of SFG sensitivity. We proceeded to show that a single *ABD1-Y416A* mutation sufficed to confer SFG resistance in the *abd1*Δ pSAM3 (*2μ HIS3 SAM3*) strain background (Fig. 3).

### *Sc*Abd1 mutations confer SFG resistance *in vitro*

We produced wild-type *Sc*Abd1 and variants L59P-Y416C, Y416N-R422H, E124K-K163R-K311R-F387Y-Y416F, and Y416A in *E. coli* as His_10_-fusion proteins and purified them by Ni-affinity and gel filtration chromatography (Supplementary Fig. S1E and F). Analytical gel filtration affirmed that they were monomeric in solution (Supplementary Fig. S1E). Thermal shift assays revealed that the thermal stability of the mutated *Sc*Abd1s (*T*_m_= 45.5°C–47.6°C) was similar to that of wild-type *Sc*Abd1 (*T*_m_= 45.3°C; Supplementary Fig. S4).

**Figure 4.**
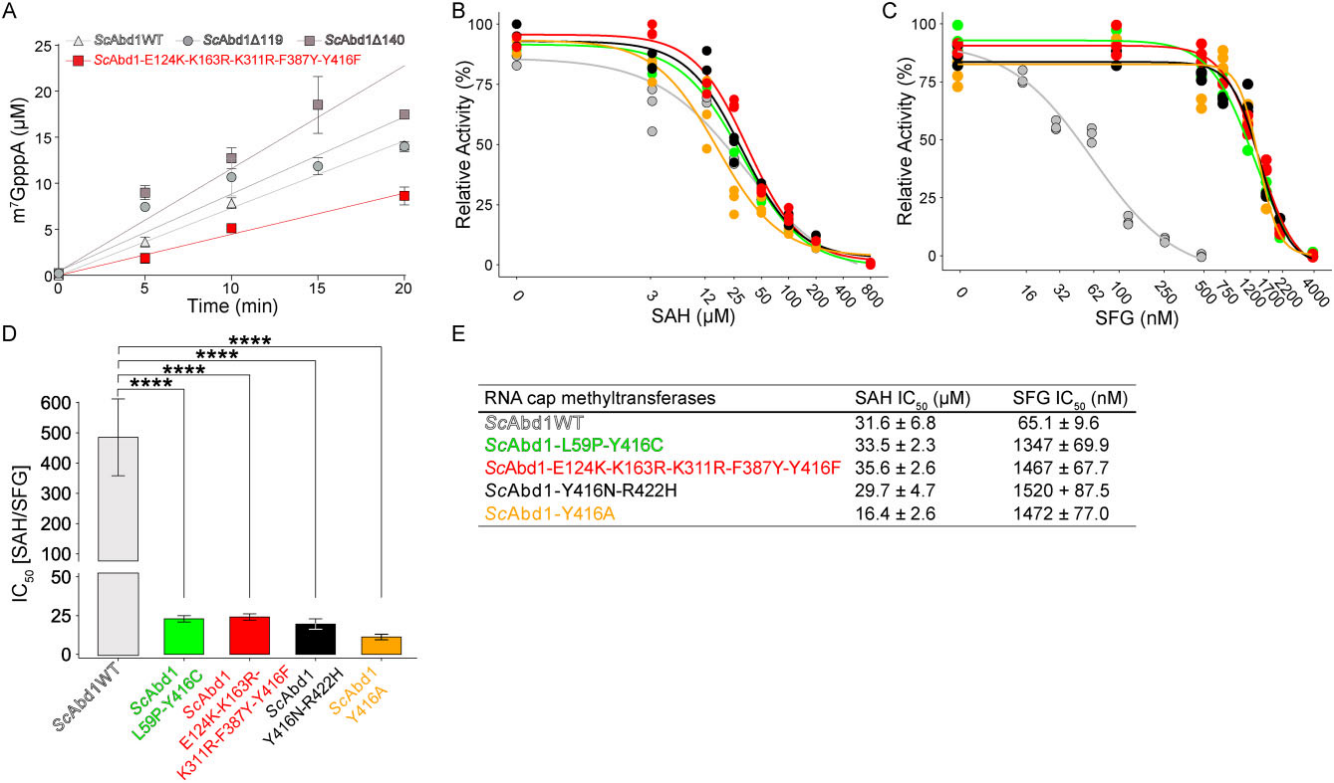
Activity and inhibition of *S. cerevisiae* Abd1. (**A**) Methylation activities of full-length wild-type (WT) *Sc*Abd1 (light gray triangles), two truncated *Sc*Abd1 variants (*Sc*Abd1Δ119, gray circles; and *Sc*Abd1Δ140, dark gray squares) and SFG-resistant *Sc*Abd1-E124K-K163R-K311R-F387Y-Y416F variant (red squares) with GpppA substrate (see the “Materials and methods” section). Error bars (one standard deviation) are calculated from three independent experiments performed in triplicate. (**B**) Inhibition of methylation activities (three independent experiments) of *Sc*Abd1WT (light gray circles), SFG-resistant variants of *Sc*Abd1 (*Sc*Abd1-L59P-Y416C, green circles; *Sc*Abd1-E124K-K163-K311R-F387Y-Y416, red circles; *Sc*Abd1-Y416N-R422H, black circles; *Sc*Abd1-Y416A, orange circles) with SAH. Reaction mixtures containing 250 nM enzyme preparations were incubated with 100 μM SAM, 250 μM GpppA, and variable concentrations (0, 3.125, 12.5, 25, 50, 100, 200, and 800 μM; see the “Materials and methods” section) of SAH at 30°C in buffer consisting of 50 mM Tris (pH 8.2), 200 mM NaCl, and 5 mM βME. After 20 min, 10 μl reaction samples were quenched with 10 μl of quenching solution containing 100 mM H_2_SO_4_ and 50 μM l-tryptophan. Concurrently, m^7^GpppA standards were quenched in quenching solution. (**C**) Inhibition of methylation activities of wild-type and SFG-resistant variants of *Sc*Abd1 with SFG as shown in panel (B). Reaction mixture containing 250 nM enzyme preparations was incubated with 100 μM SAM, 250 μM GpppA, and variable concentrations (0, 15.6, 31.25, 62.5, 125, 250, and 500 nM for the wild-type proteins; 0, 100 nM, 500 nM, 750 nM, 1.2 μM, 1.7 μM, 2.2 μM, and 4.0 μM for the variants; see the “Materials and methods” section) of SFG at 30°C in buffer consisting of 50 mM Tris (pH 8.2), 200 mM NaCl, and 5 mM βME. After 20 min, 10 μl reaction samples were quenched with 10 μl of quenching solution containing 100 mM H_2_SO_4_ and 50 μM l-tryptophan. (**D**) Ratios of IC_50_ values of wild-type and variant *Sc*Abd1 and *Kl*Abd1 between SAH and SFG with GpppA substrate. Significance between the wild-type and resistant variants determined by ordinary one-way ANOVA (**** signifies adjusted *P*-value <.0001). (**E**) IC_50_ values for SAH (μM) and SFG (nM) obtained from panels (B) and (C) for *Sc*Abd1WT (gray), *Sc*Abd1-L59P-Y416C (green), *Sc*Abd1-E124K-K163-K311R-F387Y-Y416 (red), *Sc*Abd1-Y416N-R422H (black), and *Sc*Abd1-Y416A (orange).

Enzyme titration assays showed that *Sc*Abd1 wild-type and *Sc*Abd1-E124K-K163R-K311R-F387Y-Y416F methylated cap dinucleotide GpppA with estimated turnover numbers of 3 ± 0.1 and 1.8 ± 0.1 min^−1^, respectively (Fig. 4A). Wild-type ScAbd1 and the four putative SFG-resistant variants were inhibited by SAH in a concentration-dependent manner with apparent IC_50_ values between 16.4 and 35.6 μM (Fig. 4B and E). The IC_50_ values for SAH reported here recapitulate values documented in a previous report in which methyltransferase activity was measured via a different method [7]. Wild-type *Sc*Abd1 was strongly inhibited by SFG in a concentration-dependent fashion, with an apparent IC_50_ of 65 nM (Fig. 4C), which is similar to the IC_50_ value reported previously [7]. The instructive findings here were that the four *Sc*Abd1 variants exhibited strong resistance to SFG inhibition vis-à-vis wild-type *Sc*Abd1, with apparent IC_50_ values between 1.3 and 1.5 μM (Fig. 4C and E). Therefore, whereas wild-type *Sc*Abd1 has 485-fold higher affinity for SFG than it does for SAH, the Abd1 variants have only between 11- and 24-fold higher affinity for SFG compared to SAH (Fig. 4D and E).

### Structure of *Sc*Abd1 in complex with SAH

**Figure 5.**
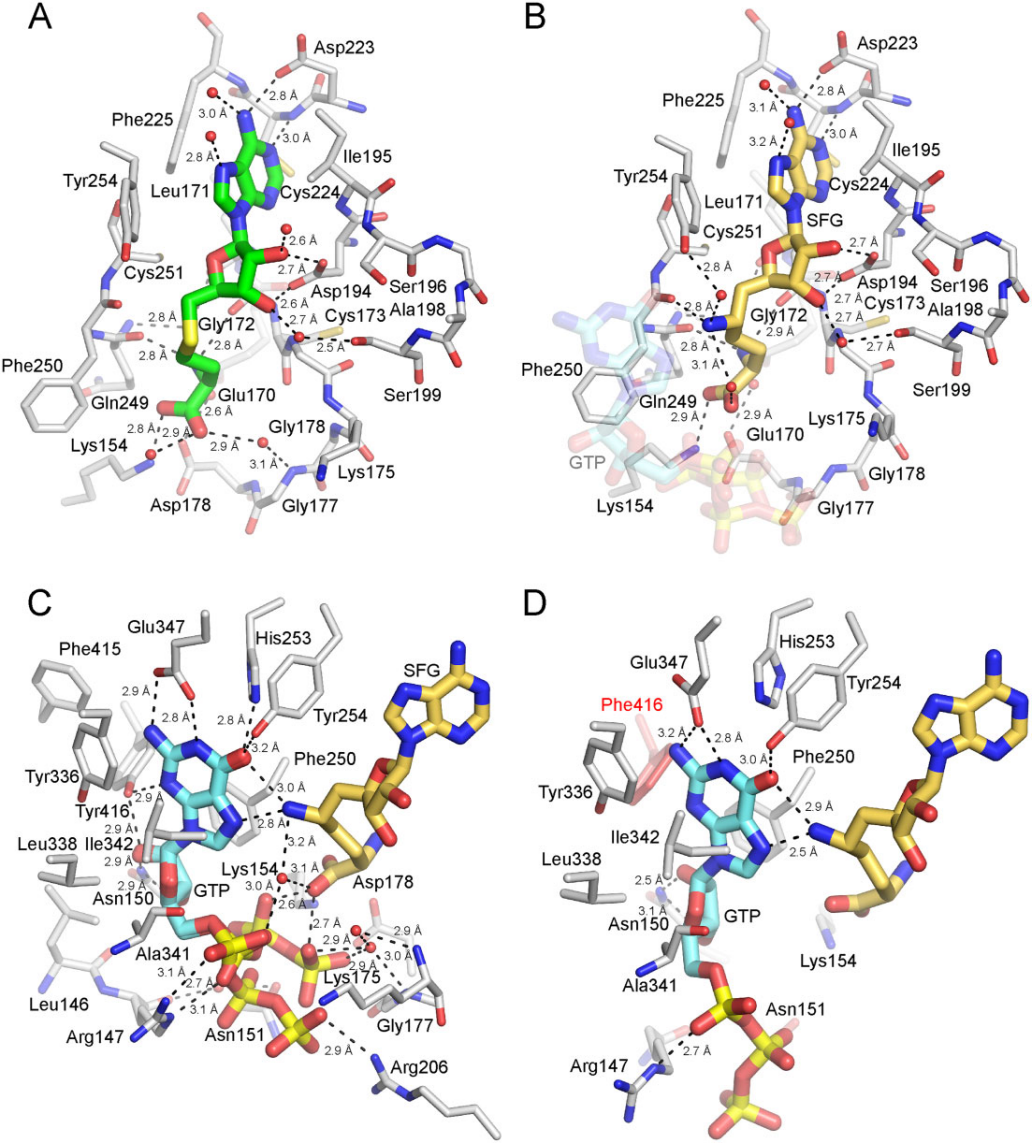
Structures of *S. cerevisiae* Abd1. (**A**) A closeup view of the methyl donor site of *Sc*Abd1 bound to SAH. (**B**) A closeup view of the methyl donor site of *Sc*Abd1 bound to SFG and GTP (transparent) as shown in stick representation and colored as in Fig. 2. (**C**) A closeup view of the methyl acceptor site of *Sc*Abd1–SFG–GTP complex. (**D**) A closeup view of the methyl acceptor site of the *Sc*Abd1Δ140-K163R-K311R-F387Y-Y416F variant in complex with SFG and GTP. Substituted Y416F side chain is colored in red. Side chains shown in stick representation (in gray), and waters are denoted by red spheres. Atomic contacts are indicated by dashed lines with distances.

Full-length wild-type *Sc*Abd1 (426-aa) did not produce diffraction quality crystals. Therefore, two N-terminal deletion variants—*Sc*Abd1Δ119 and *Sc*Abd1Δ140—were produced in *E. coli* and purified. *Sc*Abd1Δ119 and *Sc*Abd1Δ140 eluted as monomers during gel filtration (Supplementary Fig. S1C and D) and displayed *in vitro* cap methyltransferase activities (turnover numbers of 3.4 ± 0.3 min^−1^ for *Sc*Abd1Δ119 and 4.5 ± 0.4 min^−1^ for *Sc*Abd1Δ140) similar to the wild-type enzyme (Fig. 4A). We co-crystallized *Sc*Abd1Δ140 with 0.7 mM SAH and determined the structure using *Kl*Abd1Δ137•SAH as a search model. The final model was refined at 1.75 Å resolution with *R*_work_/*R*_free_ of 16.4/18.6 (Supplementary Table S1). The asymmetric unit contained one *Sc*Abd1Δ140 protomer. Electron density for SAH was observed in the methyl acceptor site (Fig. 5A and Supplementary Fig. S5A).

*Sc*Abd1Δ140 shares 83% sequence identity with *Kl*Abd1Δ137 (Fig. 1F). The two structures are superimposable with an r.m.s.d. of 1.1 Å over 289 Cα positions. All the atomic interactions of *Sc*Abd1 with SAH recapitulate the interactions observed in the methyl donor site of the *Kl*Abd1•SAH complex (Figs 1D and 5A). The C-terminal 10-aa segment of *Sc*Abd1 (aa 427–436; Fig. 1E), which is dispensable for activity [15] and which is absent in *Kl*Abd1, adopts a random coil conformation and projects away from the core methyltransferase domain (Supplementary Fig. S6).

### Structure of *Sc*Abd1Δ119 in complex with SFG and GTP

We co-crystallized a *Sc*Abd1Δ119•SFG•GTP ternary complex by preincubating the protein with 0.7 mM SFG and 1 mM GTP. The structure was refined at 1.45 Å resolution with *R*_work_/*R*_free_ of 12.7/15.2 (Supplementary Table S1). The crystal contained two protomers in the asymmetric unit. Electron densities corresponding to SFG and GTP were observed in the methyl donor and acceptor sites, respectively (Supplementary Fig. S5B). No interpretable electron density was seen for the N-terminal segment of either protomer (aa 120–140). The two protomers in the asymmetric unit are nearly identical (r.m.s.d. of 0.3 Å over 299 Cα positions) and are superimposable on the structure of *Kl*Abd1Δ137•SAH complex with an r.m.s.d. of 0.9 Å over 290 Cα and 0.8 Å over 287 Cα positions, respectively. For subsequent discussions of the *Sc*Abd1Δ119-SFG•GTP complex, we will refer to protomer A.

In the methyl donor site of *Sc*Abd1, SFG interacts with a similar ensemble of conserved residues as seen in *Kl*Abd1 (Figs 2C and 5B). In the *Sc*Abd1•SFG•GTP ternary complex, the C-NH_2_ amine of SFG makes hydrogen bonds to the guanine-O6 (3.0 Å) and -N7 (2.8 Å) atoms of GTP (Fig. 5C) and also to the backbone carbonyl of Phe250 (2.8 Å).

**Figure 6.**
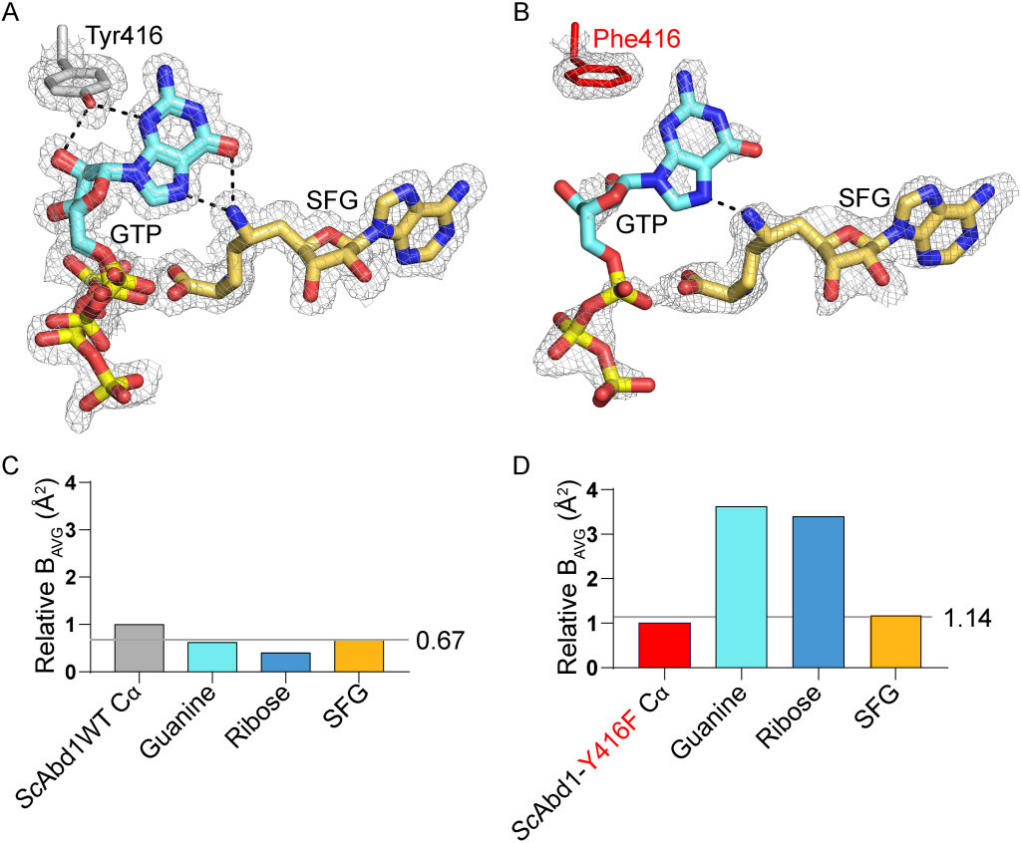
Comparison of the bound ligands in the active site of wild-type and SFG-resistant variant of *S. cerevisiae* Abd1. (**A**) A closeup view of the co-crystallized SFG and GTP in the active site of the wild-type Abd1 and the cap guanosine coordination conserved Tyr416 are shown in stick representation (colored as in Fig. 4). The gray mesh represents the model electron density (contoured at 1.0σ). (**B**) A view (as in panel A) of the bound SFG and GTP in the active site of SFG-resistant Abd1Δ140-K163R-K311R-F387Y-Y416F variant with the model electron density. Substituted Phe416 is shown in red (stick representation). Atomic contacts are indicated by dashed lines with distances. (**C**) The average *B* factor values of guanine (cyan) and ribose (light blue) of GTP and of SFG (gold) in the wild-type ternary complex were normalized to the average *B* factor value of the Cα atoms of the wild-type *Sc*Abd1 (gray) and are plotted in bar graph format. (**D**) The average *B* factor values of guanine and ribose of GTP and of SFG (colored as in panel C) in the variant ternary complex were normalized to the average *B* factor value of the Cα atoms of ScAbd1-K163R-K311R-F387Y-Y416F (red).

The GTP guanosine in the *Sc*Abd1 methyl acceptor site (Fig. 5C) interacts with a set of conserved residues analogous to the contacts observed in the *Kl*Abd1•SFG•GTP ternary complex (Fig. 2C). The electron density maps highlighted two alternate conformations for the triphosphate moiety of GTP, entailing different direct and water-mediated contact with surrounding positively charged side chains and the SFG amine and carboxylate groups (Fig. 6A and Supplementary Fig. S5B). This was the case for both *Sc*Abd1 protomers. The conformational heterogeneity of the GTP may stem from the fact that it lacks the 5′ nucleotides of GpppRNA, the physiological substrate for Abd1.

### Structure of SFG-resistant *Sc*Abd1Δ140-(K163R-K311R-F387Y-Y416F) with SFG and GTP

To elucidate the role of Tyr416 substitutions in *Sc*Abd1 in conferring SFG resistance, *Sc*Abd1Δ140-K163R-K311R-F387Y-Y416F was purified and co-crystallized in the presence of 1 mM SFG and 3 mM GTP. The structure was refined at a resolution of 2.8 Å with *R*_work_/*R*_free_ of 18.2/21.7. The crystals contained three *Sc*Abd1 complexes in the asymmetric unit and the tertiary structures of the protomers are very similar to each other (r.m.s.d. between 0.21 and 0.25 Å over 256 Cα positions). Electron densities for SFG and GTP in the methyl donor and acceptor sites were evident in all three protomers (Fig. 6B and Supplementary Fig. S5C). SFG engages with the same ensemble of enzymic residues observed in the wild-type *Sc*Abd1•SFG•GTP structure (Fig. 5B) and the C-NH_2_ amine of SFG makes hydrogen bonds with the O6 (2.9 Å) and N7 (2.5 Å) atoms of GTP, à la the wild-type structure.

Whereas the GTP contacts in the SFG-resistant *Sc*Abd1 recapitulate many of those seen in the wild-type ternary complex (Fig. 5C and D), there are some noteworthy differences. First, only one of the Glu347 carboxylate oxygens is coordinating the guanine-N1 (2.8 Å) and -N2 (3.2 Å) atoms. Second, there is no contact between His253 and guanine-O6 in the variant complex. Third, and most relevant, the Tyr416 phenolic hydroxyl-mediated polar contacts with the guanine-N3 and ribose-O2′ atoms of GTP observed in the wild-type active site are missing in the SFG-resistant variant in which Tyr416 is changed to Phe (Fig. 6A and B). Absence of the two hydrogen bonds between GTP and Tyr416 is likely to reduce the cap binding affinity of the SFG-resistant variants. Consistent with the notion, co-crystallization of the ternary complex for the variant *Sc*Abd1 required a three-fold higher concentration of GTP vis-à-vis the wild-type *Sc*Abd1 (Supplementary Table S1). In addition, whereas the electron densities for Phe416 and SFG in the resistant variant are well defined, the density for GTP is relatively weak, specifically around the guanosine and ribose moieties, when compared to the GTP density in the wild-type ternary complex (Fig. 6B and Supplementary Fig. S5C). This difference is reflected in the ∼3-fold higher average *B* factors for the GTP guanine and ribose in the variant ternary complex (normalized to that of the SFG Cα) versus the wild-type ternary complex (Fig. 6C and D).

## Discussion

### Conserved guanosine and tyrosine interactions provide a rationale for the SFG sensitivity and resistance of fungal RNA cap methyltransferases

SFG is a promiscuous inhibitor of SAM-dependent methyltransferases because it competes with SAM for binding to the methyl donor site, wherein it is unreactive with the substrate in the methyl acceptor site. The cap guanine-N7 methyltransferases of the fungi *K. lactis* and *S. cerevisiae* are highly sensitive to SFG. Whereas their IC_50_ values for SAH indicated that substrate SAM and product SAH bind to these cap methyltransferases with similar affinity (12.3 ± 4.1 and 31.6 ± 6.8 μM), their IC_50_ values for SFG are 240-fold and 485-fold lower than those for SAH (Figs 2 and 4). The structures presented here of fungal Abd1•GTP•SFG ternary complexes reveal the basis for the high potency/affinity of SFG when interdicting cap methylation, which we propose is predicated on the three unique atomic contacts made by the SFG amine, entailing (i) two hydrogen bonds to the guanine-O6 and -N7 atoms of the methyl acceptor and (ii) a hydrogen bond to a main-chain carbonyl of Abd1. In effect, SFG is rendered potent by the Abd1-bound cap guanylate substrate (i.e. substrate-assisted inhibition).

The output of a genetic selection for SFG resistance in *S. cerevisiae* linked to the *ABD1* gene converged on amino acid substitutions for Tyr416 as the common feature associated with acquired drug resistance *in vivo* and *in vitro*. Based on these results, we demonstrated that a single Y416A missense change sufficed for SFG resistance *in vivo* and *in vitro*. In both *Sc*Abd1 and *Kl*Abd1, the phenolic OH of this conserved tyrosine donates hydrogen bonds to the guanine-N3 and ribose O2′ atoms of the cap guanosine. A crystal structure of one of the SFG-resistant Abd1 enzymes (with a Phe in lieu of Tyr416) in a ternary complex with GTP and SFG highlighted that the loss of these hydrogen bonds is associated with weaker electron density and higher *B* factors for the cap guanosine vis-à-vis what is observed for the wild-type ternary complex (Fig. 6). The genetically selected SFG-resistant mutations have virtually no effect on the IC_50_ for SAH (Fig. 4E), implying that the methyl donor site is unperturbed. The IC_50_ for SFG is increased by 20-fold in the SFG-resistant mutants, presumably because of a subtle change in the position or conformational dynamics of the cap guanosine with which SFG interacts to achieve it potency.

SFG inhibits human cap guanine-N7 methyltransferase RNMT with an IC_50_ 130-fold lower than that of SAH [38]. The structure of an RNMT in a ternary complex with SFG and GppNHp (PDB 8Q9W [39]) underscores that the SFG amine participates only one hydrogen bond with the guanine-N7 atom of the methyl acceptor, and not with the guanine-O6 as observed for both *Kl*Abd1 and *Sc*Abd1 (Figsd 2D and 5C, and Supplementary Fig. S7).

### Tyr416 is conserved in the RNA cap methyltransferases of human fungal pathogens

**Figure 7.**
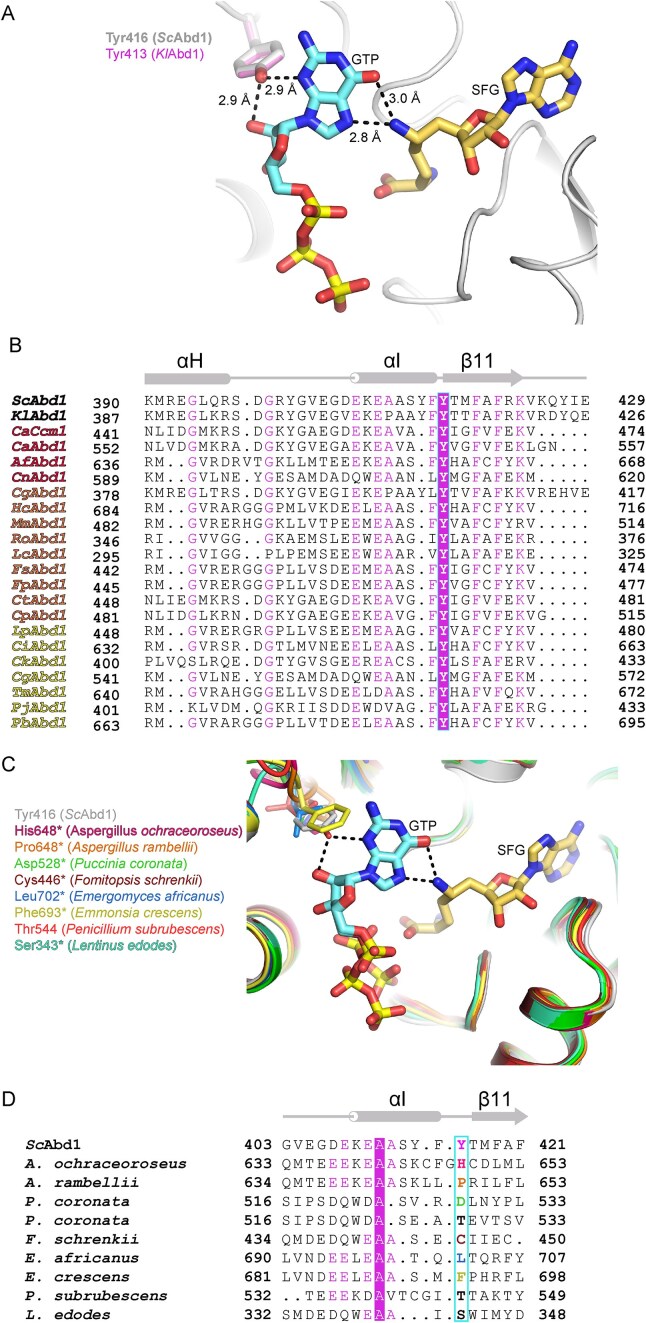
Conserved tyrosine and guanosine interaction in SFG resistance. (**A**) A closeup view of the interactions at the *Sc*Abd1 active site bound to SFG (thick transparent) and GTP (shown as in Fig. 4) highlights interactions between the conserved Tyr416, cap guanosine, and SFG. Analogous Tyr413 of *Kl*Abd1 is shown as in Fig. 1. Atomic contacts are indicated by dashed lines with distances. (**B**) Structure based aligned primary sequences of *S. cerevisiae* Abd1 (*Sc*Abd1) around Tyr416 (outlined in cyan) with orthologous RNA cap methyltransferases from *K. lactis* (*Kl*Abd1), *C. albicans* (*Ca*Ccm1; UniProt ID Q5ADX5), *C. auris* (*Ca*Abd1; UniProt ID A0A2H0ZGN4), *A. fumigatus* (*Af*Abd1; UniProt ID Q4WN42), *C. neoformans* (*Cn*Abd1; UniProt ID P0CO64), *C. glabrata* (*Cg*Abd1; UniProt ID Q6FML4), *Histoplasma capsulatum* (*Hc*Abd1; UniProt ID A0A8A1M0E7), *Madurella mycetomatis* (*Mm*Abd1; UniProt ID A0A150ASM6), *Rhizopus oryzae* (*Ro*Abd1; UniProt ID A0A9P7C5P6), *Lichtheimia corymbifera* (*Lc*Abd1; UniProt ID A0A068RQN7), *Fusarium solani* (*Fs*Abd1; UniProt ID C7YMA9), *Fusarium proliferatum* (*Fp*Abd1; UniProt ID A0A365NFV9), *Candida tropicalis* (*Ct*Abd1; UniProt ID C5MHN9), *Candida parapsilosis* (*Cp*Abd1; UniProt ID G8BB40), *Lomentospora prolificans* (*Lp*Abd1; UniProt ID A0A2N3NG19), *Coccidioides immitis* (*Ci*Abd1; UniProt ID A0A0J6Y0M7), *Candida krusei* (*Ck*Abd1; UniProt ID A0A1V2LJF6), *Cryptococcus gattii* (*Cg*Abd1; UniProt ID A0A9R1CHF2), *Talaromyces marneffei* (*Tm*Abd1; UniProt ID A0A093W0R1), *Pneumocystis jirovecii* (*Pj*Abd1; UniProt ID L0PBD4), and *Paracoccidioides brasiliensis* (*Pb*Abd1; UniProt ID A0A1D2JEX7). Sequences are shaded according to sequence conservation as in Fig. 1. Secondary structural elements of *Sc*Abd1 above the amino acid sequences. Fungal species listed in the WHO priority list as critical, high, and medium are colored as red, orange, and yellow, respectively. (**C**) Structural superimposition of the active of *Sc*Abd1 (as in panel A) with AlphaFold models (denoted by *) of *Aspergillus ochraceoroseus* (UniProt ID A0A0F8U2E0), *Aspergillus rambelli* (UniProt ID A0A0F8WQI8), *Puccinia coronata* (UniProt ID A0A2N5VA85), *Fomitopsis schrenkii* (UniProt ID S8E4P7), *Emergomyces africanus* (UniProt ID A0A1B7NRF3), *Emmonsia crescens* (UniProt ID A0A2B7ZPJ1), *Penicillium subrubescens* (UniProt ID A0A1Q5TDI6), and *Lentinus edodes* (UniProt ID A0A1Q3EMQ4) highlights natural variations at the Tyr416 position of *Sc*Abd1. (**D**) Corresponding primary sequence alignment of panel (D) around the Tyr416 (outlined in cyan) of *Sc*Abd1. Side-chain identity/similarity is denoted as in Fig. 1.

The discovery of novel antifungal agents that are active against the foremost human fungal pathogens has been designated by the World Health Organization (WHO) as a top priority for research and public health action [40]. WHO categorized the human fungal pathogens into three priority groups: critical, high, and medium. The Abd1 orthologs of the priority fungal pathogens share between 27% (*Candida glabrata*) and 44% (*C. albicans*) sequence identities with *Sc*Abd1, and the sequence alignment in Fig. 7B (with species color coded by priority class) underscores that the cap guanosine-interacting tyrosine (Tyr413 in *Kl*Abd1 and Tyr416 in *Sc*Abd1) in the methyl acceptor site is conserved in all of these fungal pathogens. Insofar as this tyrosine is a key determinant of SFG sensitivity in *Sc*Abd1, we speculate that the cap methyltransferases of the pathogenic fungi may also be highly sensitive to SFG, as defined by the criterion of a high SAH/SFG IC_50_ ratio (Fig. 4D). We note, however, that the mere presence of the conserved tyrosine is not sufficient for high SFG sensitivity. To wit, *E. cuniculi* Ecm1 has the conserved tyrosine, yet its SAH/SFG IC_50_ ratio is only 3 [11]. Biochemical, pharmacological, genetic, and structural characterization of the Abd1 orthologs of the most critical fungal pathogens (e.g. *Aspergillus fumigatus*, *Candida auris*, and *Cryptococcus neoformans* [40]) will be most valuable in consolidating the case for cap methylation as a broad antifungal therapeutic target.

### Mutations of an amino acid that dictates SFG potency are a potential source of natural fungal SFG resistance

SFG is an antibiotic produced by *Streptomyces griseolus*, a soil-dwelling filamentous bacteria indigenous to Côte d’Ivoire [41]. Soil communities, which are rich in microbial diversity, engage in intense competition for resources. Whole genome sequencing results of Côte d’Ivoire soil communities reveal sequences of putative fungal RNA cap methyltransferases from *A. ochraceoroseus* (UniProt ID A0A0F8U2E0) and *A. rambellii* (UniProt ID A0A0F8WQI8), and the corresponding AlphaFold models [42] indicate that these sequences adopt structures closely related to yeast Abd1 (Fig. 7C and D). Comparison of these modeled methyltransferase structures with that of *Sc*Abd1 reveals that in the *A. ochraceoroseus* and *A. rambellii* RNA cap methyltransferase models the amino acid analogous to *Sc*Abd1 Tyr416 is replaced by non-tyrosine side chains: His648 in *A. ochraceoroseus* and Pro648 in *A. rambellii* (Fig. 7D). In contrast, most of the RNA cap methyltransferases in *Aspergillus* species that are not indigenous to Côte d’Ivoire contain a tyrosine at this position (Supplementary Fig. S8). Therefore, it is conceivable that these tyrosine substitutions potentially afford a selective advantage for *A. ochraceoroseus* and *A. rambellii*, which share a common habitat with SFG producing *S. griseolus*. It will be of interest to interrogate the SFG sensitivity of Abd1 enzymes from *Aspergillus* species that have or do not possess the conserved tyrosine.

The mining of AlphaFold models of fungal Abd1 homologs revealed additional taxa in which the amino acid equivalent of *Sc*Abd1 Tyr416 is not conserved (Fig. 7C and D), instead being replaced by (i) an aspartate or a threonine in *P. coronata* strain 12SD80 (UniProt ID A0A2N5VA85) and strain 12NC29 (UniProt ID A0A2N5VJX1), respectively, (ii) a cysteine in *F. schrenkii* (UniProt ID S8E4P7), (iii) a leucine in *E. africanus* (UniProt ID A0A1B7NRF3), (iv) a phenylalanine in *E. crescens* strain UAMH4076 (UniProt ID A0A2B7ZPJ1), (v) a threonine in *P. subrubescens* (UniProt ID A0A1Q5TDI6), and (vi) a serine in *L. edodes* (UniProt ID A0A1Q3EMQ4). With the limited availability of whole genome sequence information for the ecological niches of these organisms, it is not possible to predict the selective pressures that are encountered.

### SFG as a plausible scaffold for design of inhibitors of fungal RNA cap methyltransferases

SFG has several characteristics that advocate for its potential in antifungal drug design. First, exogenous SFG is taken up across the plasma membrane by a high affinity yeast-transporter Sam3, the same protein that takes up exogenous SAM [26, 37]. In contrast, neither SAM nor SFG is efficiently taken up by mammalian cells. Second, SFG clearly “hits” its target Abd1 *in vivo*, as elucidated in this study, at least in budding yeast as a model fungus. We envision that selectivity for interdicting Abd1, and avoidance of targeting other methyltransferases, might be enhanced by the design of SFG-based bisubstrate analogs that simultaneously fill the methyl donor and cap acceptor sites of the Abd1 orthologs.

## Supplementary Material

gkaf538_Supplemental_Files

## Data Availability

Structure factors and coordinates of Abd1 orthologs and variant are deposited in the RCSB Protein Data Bank with accession codes 9MG0 (*Kl*Abd1Δ137•SAH), 9MG1 (*Kl*Abd1Δ137•m^7^GTP), 9MG2 (*Kl*Abd1Δ137•SFG), 9MG3 (*Kl*Abd1Δ137•SFG•GTP), 9MG4 (*Sc*Abd1Δ140•SAH), 9MG5 (*Sc*Abd1Δ119•SFG•GTP), and 9MG6 (*Sc*Abd1Δ140-K163R-K311R-F387Y-Y416F•SFG•GTP).
