# Supplementary material for: Structural basis for sensitivity and acquired resistance of fungal cap guanine-N7 methyltransferases to the antifungal antibiotic sinefungin
Source: Nucleic Acids Res. 2025 Jul 19;53(14):gkaf538. doi: 10.1093/nar/gkaf538 (PMC12276006; doi:10.1093/nar/gkaf538)
Supplement: gkaf538_Supplemental_Files [file gkaf538_supplemental_files.zip › abd1-SFG_updated_SI.pdf]

Supporting Information

Structural basis for sensitivity and acquired resistance of fungal cap guanine-N7 methyltransferases to the antifungal antibiotic sinefungin

Daniel J. Nilson[1], Beate Schwer[3], Steven C. Almo[1], Stewart Shuman[2] and Agnidipta Ghosh[1]*

[1]Department of Biochemistry, Albert Einstein College of Medicine, 1300 Morris Park Avenue Bronx, NY 10461; [2]Molecular Biology Program, Memorial Sloan Kettering Cancer Center, New York, NY 10021; [3]Department of Microbiology and Immunology, Weill Cornell Medical College, New York, NY 10021

*Corresponding author: agnidipta.ghosh@einsteinmed.edu

Table of contents

Figure S1: Purification of fungal RNA cap methyltransferases 2
Figure S2: Structural comparisons of *Kl*Abd1Δ137 with Ecm1 and RNMT 3
Figure S3: Electron densities of active site ligands in *Kl*Abd1Δ137 structures....4
Figure S4: Stability of wild-type and SFG resistant variant of *Sc*Abd1 5
Figure S5: Active site of wild-type and SFG resistant variant of *Sc*Abd1 bound to SAH and SFG and GTP....6
Figure S6: Most of the substituted sidechains in SFG resistant variants are distal to the Abd1 active site....7
Figure S7: Comparison of bound ligands in the active site of *Kl*Abd1, *Sc*Abd1 and RNMT 7
Figure S8: Conserved cap guanosine coordinating tyrosine in *Sc*Abd1 and *Aspergillus* species....8
Table S1: Crystallization conditions, crystallographic data statistics and RCSB IDs 9
References 9

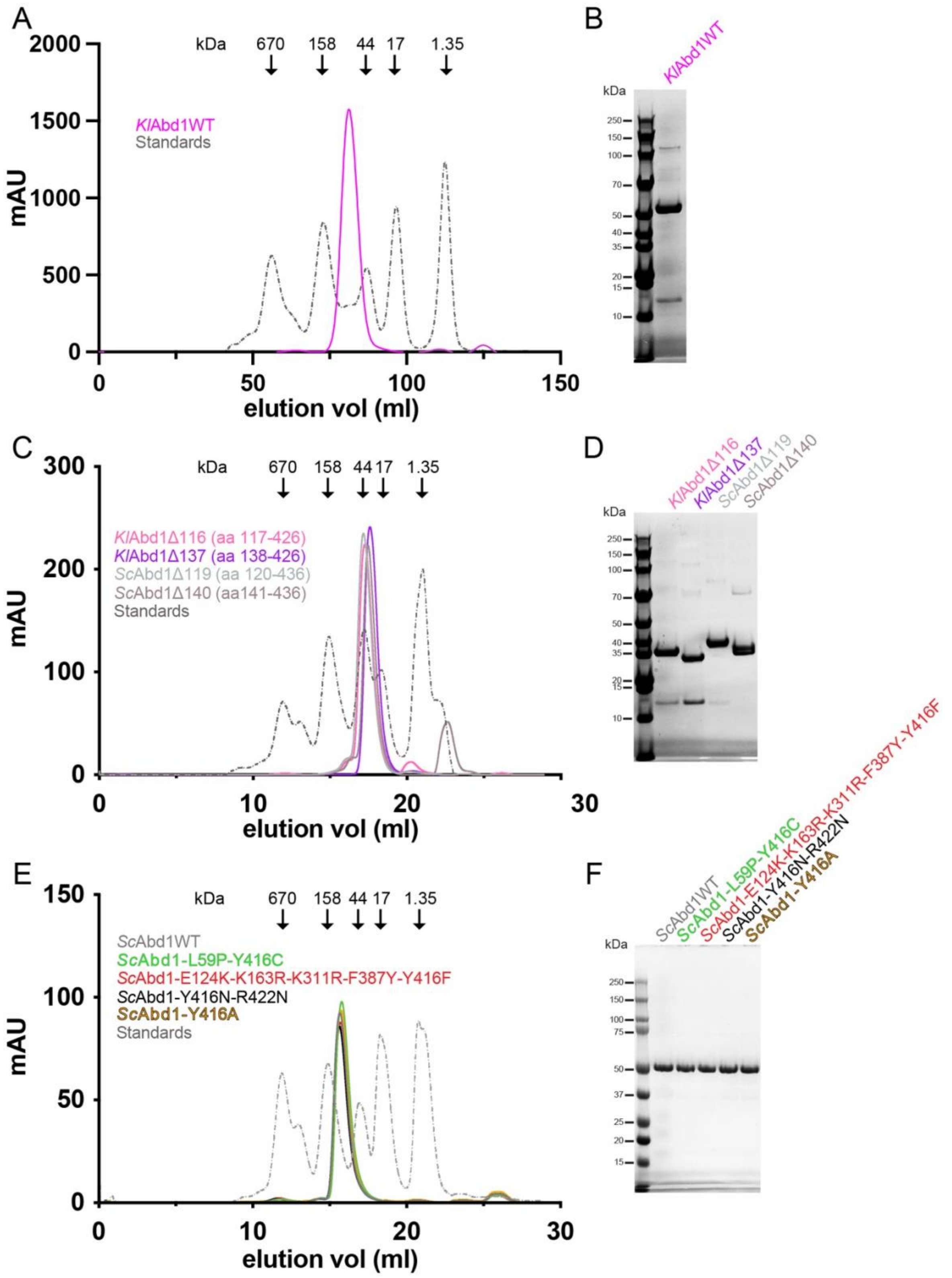

Figure S1. Purification of fungal RNA cap methyltransferases. (**A**) The Superdex S200 analytical gel filtration elution profile of full-length wild-type (WT) *Kl*Abd1 (purple line) is shown.

The dotted grey line shows the elution profile of molecular weight standards standards (kDa, denoted by arrows). (**B**) An aliquot (5 µg) of the peak Superdex S200 *Kl*Abd1WT fraction was analyzed by SDS-PAGE. The Coomassie blue stained gel is shown. The sizes (kDa) of marker polypeptides analyzed in parallel are indicated on the left. (**C**) The Superdex S200 elution profiles of *Kl*Abd1Δ116 (pink), *Kl*Abd1Δ137 (dark purple), *Sc*Abd1Δ119 (grey) and *Sc*Abd1Δ140 (dark grey) are shown. (**D**) Aliquots (5 µg) of the peak Superdex S200 fractions of the indicated methyltransferases were analyzed by SDS-PAGE. (**E**) The Superdex S200 elution profiles of *Sc*Abd1WT (light grey) and SFG-resistant variants, *Sc*Abd1-L59P-Y416C (green), *Sc*Abd1-E124K-K163R-K311-R-F387Y-Y416F (red), *Sc*Abd1-Y416N-R422N (black) and *Sc*Abd1-Y416A (orange) are shown. (**F**) Aliquots (4 µg) of the peak Superdex S200 fractions of wild-type *Sc*Abd1 and SFG-resistant variants were analyzed by SDS-PAGE.

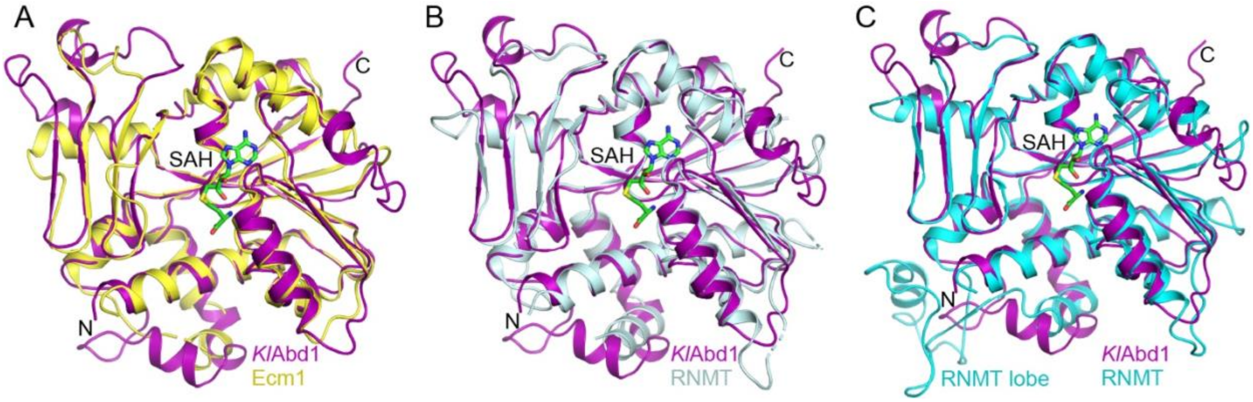

Figure S2. Structural comparisons of *Kl*Abd1Δ137 with Ecm1 and RNMT. (**A**) Supreimposition of the structures of Ecm1 (in yellow) and SAH-bound *Kl*Abd1Δ137 (in purple). SAH is shown in stick representation with green carbons. **B**) Supreimposition of the structures of RAM-free RNMT (PDB 3BGV; protomer A; colored aquamarine) and *Kl*Abd1Δ137 (purple). **C**) Supreimpostion of the structures of RAM-bound RNMT (PDB 5E8J; protomer A; colored cyan) and *Kl*Abd1 (purple). An RNMT-specific lobe comprising two β strands (β10a and β10b) and an α helix is indicated at bottom left.

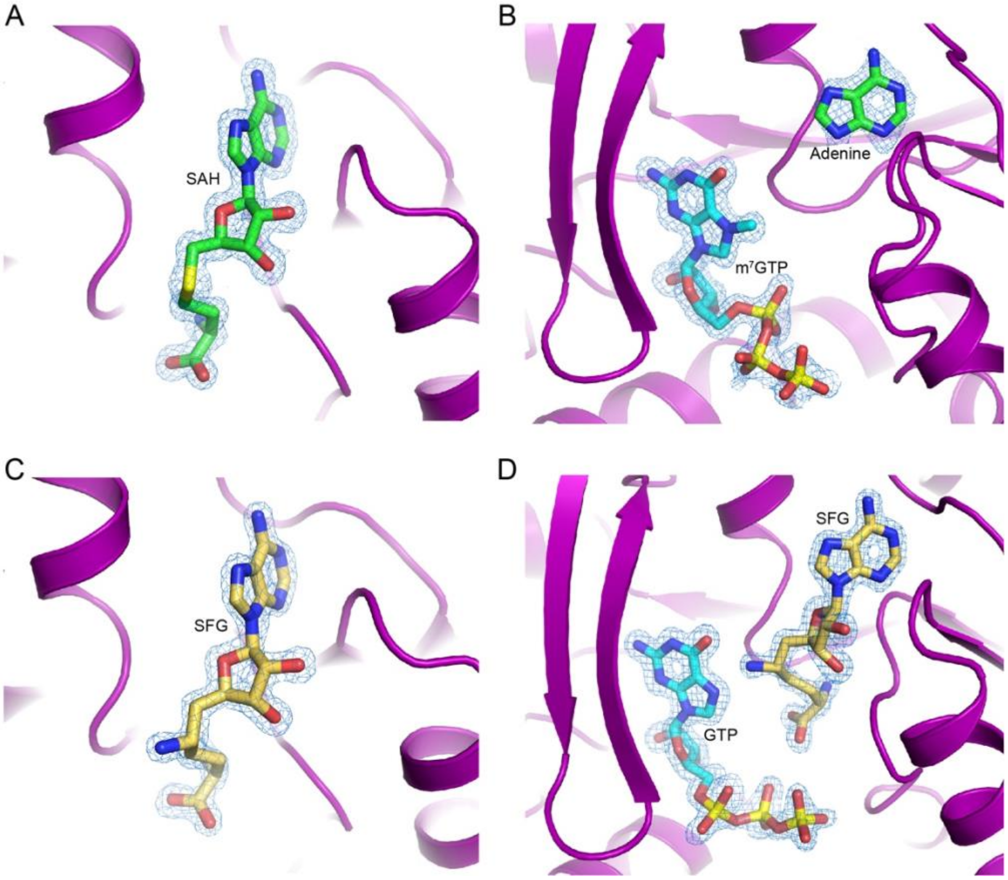

Figure S3. Electron densities of active site ligands in *Kl*Abd1Δ137 structures. (**A**) Omit map (blue mesh; contoured at 3.0 σ) corresponding to SAH (stick model with green carbons) in the active site of *Kl*Abd1Δ137. (**B**) Omit maps (contoured at 2.5 σ) corresponding to adenine (stick model with green carbons) and m^7GTP (stick model with cyan carbons) in the active site of *Kl*Abd1Δ137. (**C**) Omit maps (contoured at 2.5 σ) corresponding to SFG (stick model with gold carbons) in the active site of *Kl*Abd1Δ137. (**D**) Omit maps (contoured at 2.5 σ) corresponding to SFG (gold carbons) and GTP (cyan carbons) in the active site of *Kl*Abd1Δ137.

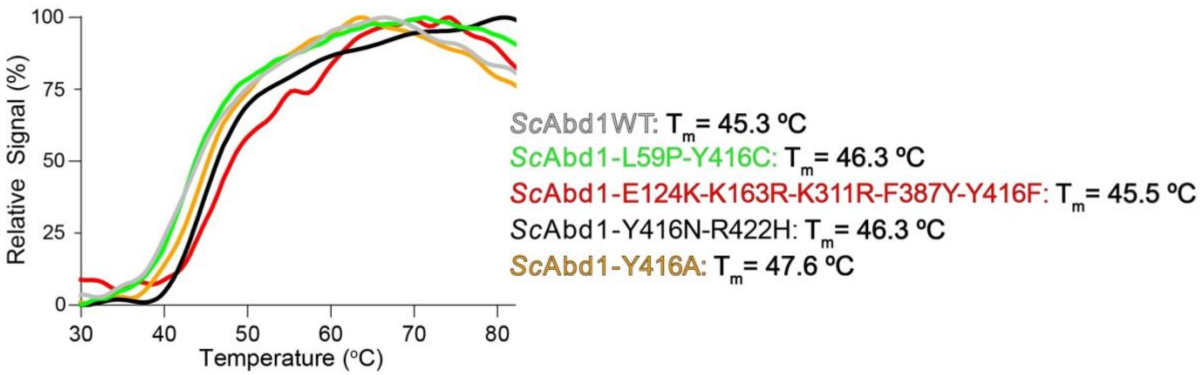

Figure S4. Stability of wild-type and SFG resistant variant of *Sc*Abd1. Differential scanning fluorimetry of wild-type and SFG resistant variants of *Sc*Abd1. Denaturation profiles of *Sc*Abd1WT (light grey), *Sc*Abd1-L59P-Y416C (green), *Sc*Abd1-E124K-K163-K311R-F387Y-Y416 (red), *Sc*Abd1-Y416N-R422H (black) and *Sc*Abd1-Y416A (orange) as a function of temperature as observed by increase in fluorescence of the indicator dye SYPRO Orange, which binds nonspecifically to hydrophobic surfaces of unfolded proteins. At higher temperatures, the intrinsic fluorescence degrades due to the formation of protein aggregates and dye dissociation. Corresponding melting temperatures (T_m) for the wild-type and variant ScAbd1 are indicated on the right.

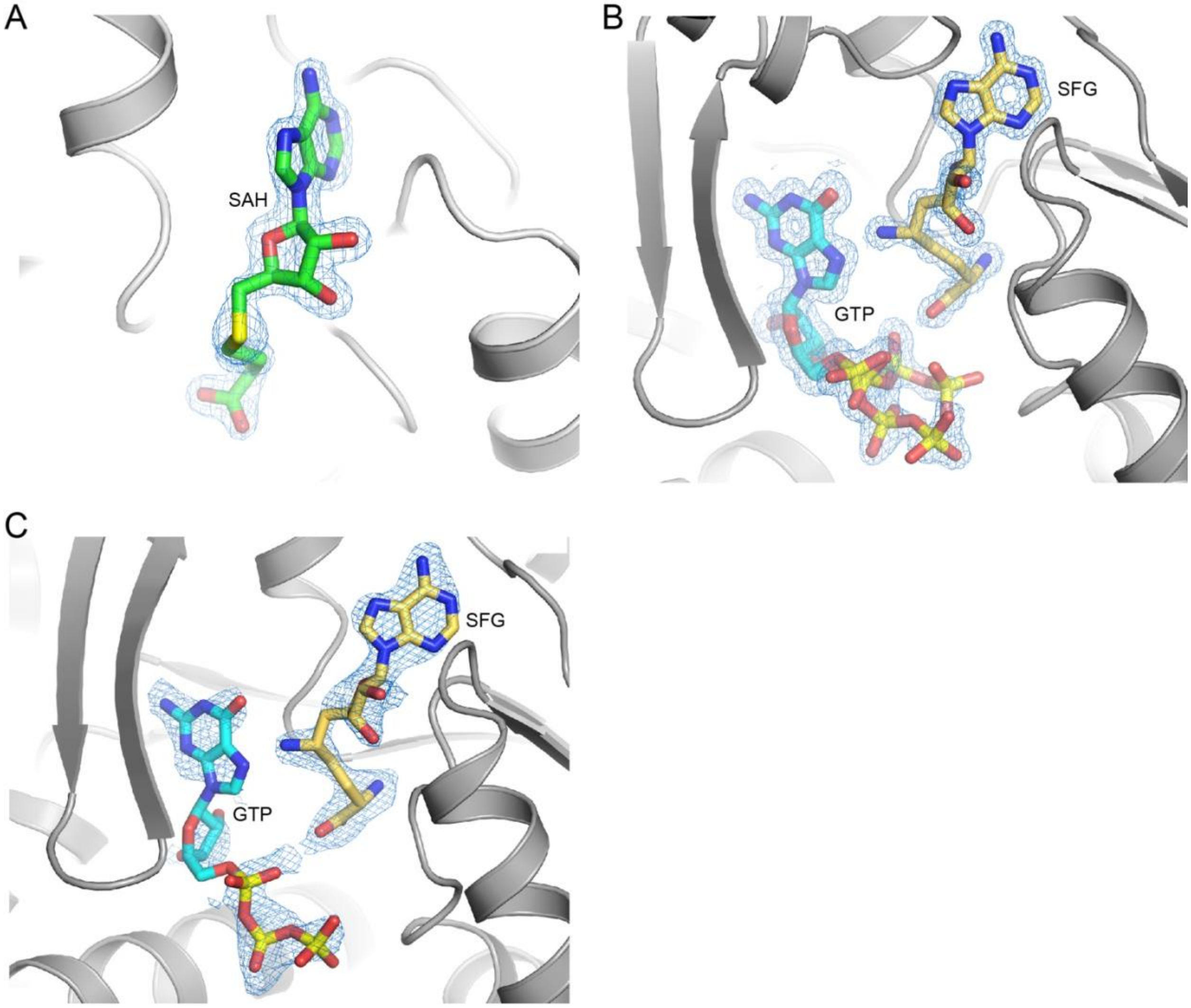

Figure S5. Active site of wild-type and SFG resistant variant of *Sc*Abd1 bound to SAH and SFG and GTP. A) Omit maps (blue mesh; contoured at 2.5 σ) corresponding to bound SAH in the active site of *Sc*Abd1Δ140-WT. **B**) Omit maps (contoured at 2.5 σ) corresponding to bound SFG and GTP in the active site of *Sc*Abd1Δ119-WT (protomer A). **C**) Omit maps (contoured at 2.0 σ) corresponding to bound SFG and GTP in the active site of the SFG resistant variant *Sc*Abd1Δ140-K163R-K311R-F387Y-Y416F (protomer A).

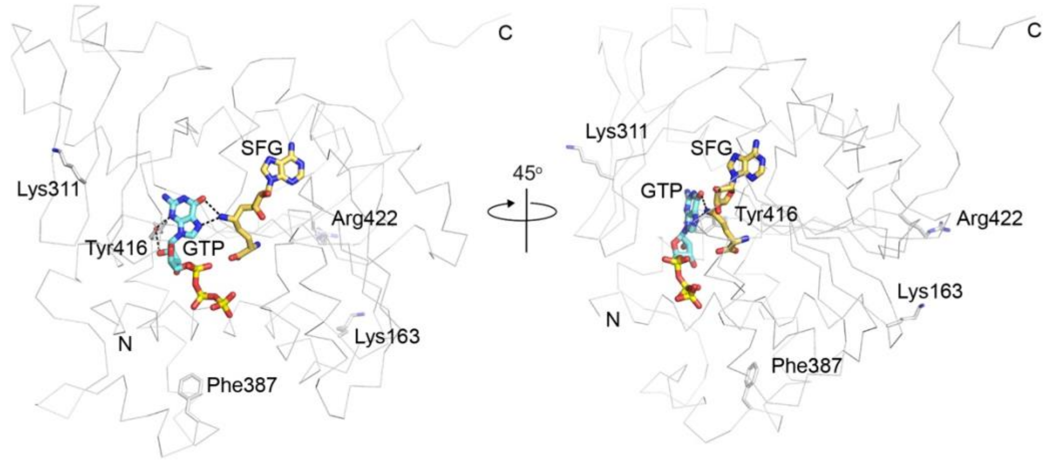

Figure S6. Most of the substituted sidechains in SFG resistant variants are distal to the Abd1 active site. A) Views highlight amino acid positions (shown as in Figure 4) identified in the *Sc*Abd1 (in light grey ribbon) SFG-resistant strains (Figure 3). Atomic contacts are indicated by dashed lines.

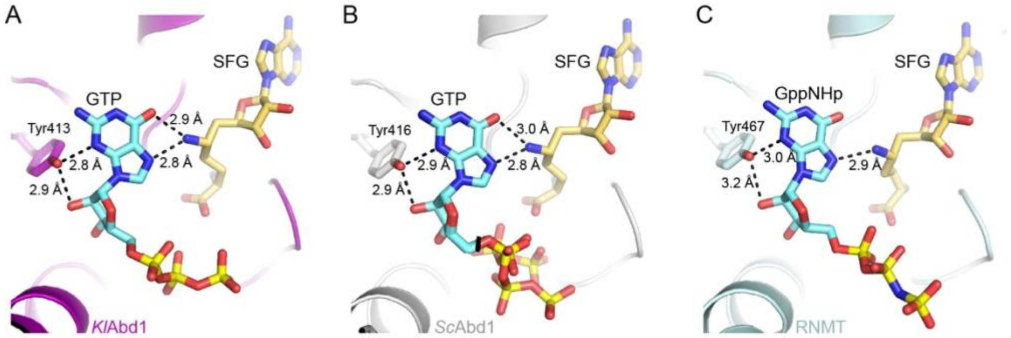

Figure S7. Comparison of bound ligands in the active site of *Kl*Abd1, *Sc*Abd1 and RNMT. A) A closeup view of the interactions at the *Kl*Abd1 (PDB 9MG3) active site bound to SFG and GTP (shown as in Figure 2) highlights interactions between the conserved Tyr413, cap guanosine and SFG. **B**) SFG and GTP bound active site and Tyr416 of *Sc*Abd1 (PDB 9MG5) is shown as in Figure 5. C) A closeup view of the active site of RNMT (as in Figure S2; PDB 8Q9W) highlighting interactions of Tyr467 with bound to SFG and GppNHp. Atomic contacts are indicated by dashed lines with distances.

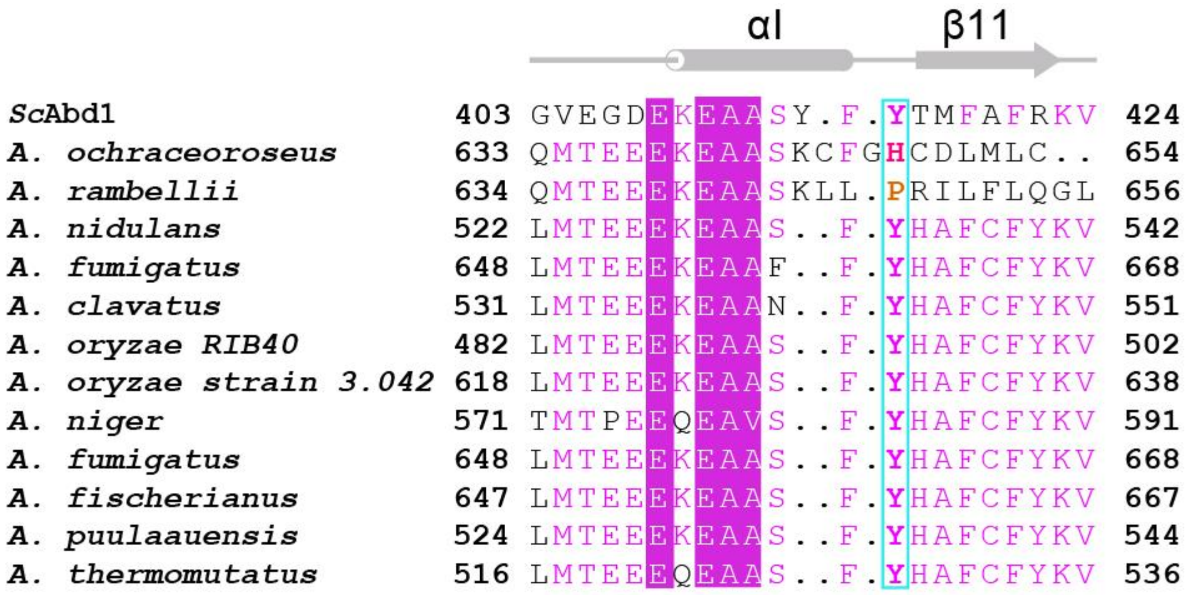

Figure S8: Conserved cap guanosine coordinating tyrosine in *Sc*Abd1 and *Aspergillus* species. Primary structure alignment of *Sc*Abd1 around Tyr416 (outlined in cyan) with RNA cap methyltransferases from *Aspergillus* species, *A. ochraceoroseus* (Uniprot A0A0F8U2E0), *Aspergillus rambelli* (Uniprot A0A0F8WQI8), *A. nidulans* (Uniprot C8VKT7), *A. fumigatus* strain ATCC MYA-4609 (Uniprot Q4WN42)*, A. clavatus* (Uniprot A1CT57), *A. oryzae RIB40* (Uniprot Q2UM19), *A. oryzae strain 3.042* (Uniprot I7ZMY1), *A. niger* (Uniprot G3YCD0), *A. fumigatus* (strain CBS 144.8; Uniprot B0Y7F7), *A. fischerianus* (Uniprot A1DMG9), *A. puulaauensis* (Uniprot A0A7R7XG76) and *A. thermomutatus* (Uniprot A0A397HLN8). Sidechain identity/similarity is denoted as in Figure 1. Secondary structure elements (in light grey) of *Sc*Abd1 are above the sequence.

Table S1: Crystallization conditions, crystallographic data statistics and RCSB IDs

**Protein**	*Kl* Abd1Δ137	*Kl* Abd1Δ137	*Kl* Abd1Δ137	*Kl* Abd1Δ137	*Sc* Abd1Δ140	*Sc* Abd1Δ119	*Sc* Abd1Δ140-K163R-K311R-F387Y-Y416F
**Ligand**$^{\$}$	0.7 mM SAH	0.9 mM m^7GTP	0.6 mM SFG	0.6 mM SFG and 1 mM GTP	0.7 mM SAH	0.7 mM SFG and 1 mM GTP	1 mM SFG and 3 mM GTP$^{\#}$
**Crystallization condition**	0.2 M Calcium Chloride, 20% (w/v) PEG 3350, and 0.1 M Potassium Sodium Tartrate	0.2 M Lithium Sulfate, 25% (w/v) PEG 3350, and 0.1 M Bis-Tris pH 5.5	0.2 M Magesium Chloride, and 25% (w/v) PEG 3350	0.18 M Lithium Sulfate, 29.5% (w/v) PEG 4000, and 0.1 M Tris pH 8.5	0.8 M Sodium Phosphate, 1.2 M Potassium Phosphate, and 0.1 M Sodium Acetate pH 4.5	0.2 M Ammonium Sulphate, and 20% (w/v) PEG 3350	2 M Ammonium Sulphate, 1.8% Ethylene Glycol, 100 mM Tris pH 8.5, 10 mM Tris(2-carboxyethl)phosphine hydrochloride
**Cryo preservation**	12% (v/v) Ethylene Glycol	10% (v/v) Ethylene Glycol	10% (v/v) Ethylene Glycol	10% (v/v) Ethylene Glycol	20% (v/v) Ethylene Glycol	15% (v/v) Ethylene Glycol	20% (v/v) Ethylene Glycol
**Data Collection***							
Source	BNL X29A	BNL X29A	BNL X29A	BNL X29A	BNL X29A	APS ID-31	BNL 17-ID-1
Wavelength (Å)	1.075	1.075	1.075	1.075	0.98	0.98	0.92
Number of crystals	1	1	1	1	1	1	1
Space group	$P2_1$	$P2_1$	$P2_1$	$P2_12_12_1$	$R3_2$	$P6_3$	$C222_1$
Cell dimensions							
a,b,c (Å)	63.6, 64.6, 73.5; β = 94.5°	63.2, 65.2, 74.9; β = 94.4°	45.96, 54.79, 112.93; β = 92.8°	65.1, 92.6, 101.3	95.2, 95.2, 248.9; γ = 120°	95.7, 95.7, 129.3	113.8 201.2 160.5
Resolution (Å)	2 - 1.31 (1.31 - 1.30)	24.91 - 1.33 (1.34 - 1.33)	24.64 - 1.59 (1.61 - 1.59)	39.96 - 1.42 (1.44 - 1.42)	24.83 - 1.75 (1.82 - 1.75)	29.87 - 1.45 (1.47 - 1.45)	17.98 - 2.8 (2.9 - 2.8)
Completeness (%)	98.3 (89.7)	99 (97.5)	96.4 (91.9)	99.3 (93.6)	100 (100)	99.9 (85.2)	99 (99)
Total reflections	959938	821130	337172	576584	1275413	1336800	345592 (35690)
Unique reflections	155084	137174	67646	143920	43942	183124	45455 (4499)
Wilson B-factor	11.6	12	14.61	14.4	15.42	9.7	35.98
Multiplicity	6.2 (5.7)	6.7 (5.9)	6.0 (5.4)	7.3 (4.9)	29.0 (27.2)	17.5 (15.1)	7.6 (7.9)
R_{merge} (%)	14.3 (63.8)	18.2 (51.3)	13.9 (45.5)	6.4 (56.7)	17.9 (58.3)	11.5 (78.3)	21.8 (83.7)
$CC_{1/2}$ (%)	98.5 (99.3)	95.5 (98.9)	89.0 (78.3)	99.0 (100)	99.0 (100)	99.9 (85.3)	98.5 (79.6)
CC* (%)	97.4 (97.6)	95.1 (97.2)	96.5 (87.4)	98.3 (98.0)	98.3 (98.0)	98.7 (84.2)	99.6 (94.2)
<I>/s(I)	5.5 (2.1)	3.48 (1.3)	8.6 (2.2)	17.6 (2.3)	5 (2.5)	6.3 (1.7)	8.64 (2.42)
**Refinement***							
Reflections: work/free	143738 (4333)/7303 (203)	137084 (3266)/6782 (174)	67657 (2767)/3363 (141)	114206 (3391)/ 5644(174)	43911 (2708)/2279 (142)	111696 (7825)/5934 (454)	45393 (4499)/ 2269 (235)
R_{work}/R_{free} (%)	16.6 (30.5)/18.6 (31.9)	18.5 (27.3)/20.2 (31.4)	18.6 (25.1)/21.8 (30.3)	17.5 (23.0)/20.0 (25.6)	16.5 (21.3)/18.6 (24.7)	13.0 (25.1)/15.0 (29.0)	18.3 (27.1) / 21.7 (29.8)
Number of TLS groups	18		14	8		12	3
Number of atoms							
Protein	4904	4832	4740	4798	2487	4946	7154
Ligand	68	151	105	169	16	381	258
Water	580	596	404	507	313	504	243
Average B-factors ($Å^2$)							
Protein	20.9	20.03	19.4	22.9	19.6	17.4	34.17
Ligand	20	31.6	19.3	21.7	34.66	27.1	71.26
Water	33.9	27.1	23.6	28.5	27.9	28.3	28.11
r.m.s.d.							
Bond lengths (Å)	0.01	0.01	0.01	0.01	0.01	0.02	0.009
Bond angles (°)	0.9	0.95	0.9	0.9	1.08	1.62	1.75
Molprobity†							
Favored	98.4% (569 aa)	98.4% (568 aa)	97.9% (564 aa)	97.5% (568 aa)	97.5% (291 aa)	98% (579 aa)	97.2% (859 aa)
Allowed	100% (578 aa)	100% (577 aa)	100% (576 aa)	100% (577 aa)	100% (297 aa)	100% (591 aa)	100% (879 aa)
Outliers	none	none	none	none	none	none	0.0%
Clash score	100^{hth} percentile	100^{hth} percentile	100^{hth} percentile	100^{hth} percentile	100^{hth} percentile	100^{hth} percentile	100th percentile
Molprobity score	100th percentile	100th percentile	100th percentile	100th percentile	100th percentile	100th percentile	100th percentile
**RCSB ID**	9MG0	9MG1	9MG2	9MG3	9MG4	9MG5	9MG6

$^{\$}$ Co-crystallized

* Statistics calculated using PHENIX (1); highest resolution shells indicated in parentheses

† Calculated with the program MOLPRROBITY (2)

$^{\#}$ Crystals were soaked with 3 mM GTP

References

1. Liebschner, D., Afonine, P.V., Baker, M.L., Bunkoczi, G., Chen, V.B., Croll, T.I., Hintze, B., Hung, L.W., Jain, S., McCoy, A.J. *et al.* (2019) Macromolecular structure determination using X-rays, neutrons and electrons: recent developments in Phenix. *Acta Crystallogr D Struct Biol*, **75**, 861-877.
2. Williams, C.J., Headd, J.J., Moriarty, N.W., Prisant, M.G., Videau, L.L., Deis, L.N., Verma, V., Keedy, D.A., Hintze, B.J., Chen, V.B. *et al.* (2018) MolProbity: More and better reference data for improved all-atom structure validation. *Protein Sci*, **27**, 293-315.
